# The Last Frontiers and Challenges in Urea Electrochemical Production Using Carbon Dioxide and Nitrate

**DOI:** 10.1002/cssc.70727

**Published:** 2026-05-24

**Authors:** Alessandro Bani, Mohsin Muhyuddin, Piercarlo Mustarelli, Veronica Termopoli, Rosanna Viscardi, Carlo Santoro

**Affiliations:** ^1^ Department of Materials Science University of Milano‐Bicocca Milan Italy; ^2^ Milano Chemometrics and QSAR Research Group Department of Earth and Environmental Sciences University of Milano‐Bicocca Milano Italy; ^3^ ENEA Department of Energy Technologies and Renewable Sources Casaccia Research Center Rome Italy

**Keywords:** detection systems, electrocatalysts, reaction mechanisms, urea electrosynthesis

## Abstract

To reach complete decarbonization by 2050, much effort must be devoted to the electrification of hard‐to‐abate sectors and the substitution of fossil fuels with renewable sources. Currently, one of the most polluting sectors is the ammonia production through the Haber–Bosch process which is energy‐intensive. Moreover, the hydrogen needs to produce ammonia derived from steam reforming of methane gas and the water shift reaction, in turn consuming fossil fuels and emitting more than 2% of the CO_2_ globally. Ammonia is the building block to produce urea which is the most used nitrogen‐containing fertilizer. In the past few years, urea electrosynthesis starting from CO_2_ and nitrate has captured significantly the interest of the scientific community as it could become the cornerstone to achieve an intense and resilient urea production to reduce emissions and avoid usage of fossil fuels. Here, we discuss the status of the latest achievements in this field, focusing on the reaction mechanisms, different types of electrocatalysts and catalysis pursued, and the detection of urea and their intermediates using a plethora of diverse instruments and methods. This review points out the most critical aspects for research and highlights the potential routes for overcoming the main issues to be solved.

## Graphical abstract

Urea electrosynthesis from CO_2_ and nitrate offers a sustainable alternative to fossil‐fuel‐based production. This review examines recent progress in reaction mechanisms, electrocatalysts, and detection methods for urea and intermediates. By identifying critical research challenges, we highlight potential strategies to optimize efficiency and overcome current technical barriers, aiming for resilient, low‐emission urea manufacturing.

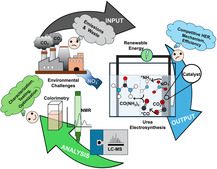

## 1 Introduction

Nearly 50% of the nitrogen atoms present in the human body come from the Haber–Bosch process [1]. This revolutionary industrial process converts hydrogen (H_2_) and nitrogen (N_2_) into ammonia, which can subsequently be transformed into a wide range of nitrogen‐based fertilizers [2]. These fertilizers are extensively utilized in agriculture worldwide and the nitrogen they contain is ultimately incorporated in the human body through food consumption. Despite the Haber–Bosch process having effectively increased food production for humanity, it is not without significant drawbacks. It is highly energy demanding because it is conducted at high temperatures and pressures (around 400°C–450°C and 150–250 atm) [3]. Moreover, H_2_ is generally produced by steam methane reforming (SMR), increasing the carbon footprint of the overall process. Harsh operating conditions and H_2_ production derived mainly from fossil fuels lead to an overall emission of 1.9 tons of CO_2_ per ton of ammonia produced, contributing to the climate change issue [4]. Among all the various nitrogen‐based fertilizers, urea is the most widespread for agricultural purposes due to its high nitrogen content and safety when handled [5]. Urea is currently produced by the Bosch–Meiser process, which exploits the reaction between ammonia and carbon dioxide at around 180–200°C and 150–200 atm [6]. The Bosch–Meiser process is energy‐intensive, CO_2_‐emissive and very expensive. From this side, the urea cost reached its maximum in 2022 (≈1000 USD per ton), inflated by the rising cost of natural gas because of the Russia–Ukraine conflict [7]. The environmental and monetary price of the Haber–Bosch process, coupled with the Bosch–Meiser process, makes urea production unsustainable.

Urea electrosynthesis emerged approximately 30 years ago in the pioneering works of Shibata [8, 9] as a promising technology to enable green urea production, coupled with renewable electricity utilization. Compared to the Bosch–Meiser process, urea electrosynthesis operates under significantly milder conditions (room temperature and pressures) and the direct use of electrons as reducing agents. Generally, carbon dioxide and a nitrogen‐containing molecule of inorganic nature are solubilized in a liquid electrolyte and subjected to a cathodic reaction on an active electrocatalyst. The electrocatalyst must be able to generate suitable adsorbed intermediates, which then bind together in the C—N coupling steps, namely, the binding process of carbon and nitrogen, which is crucial for urea electrosynthesis. The utilization of carbon dioxide as a carbon precursor is beneficial because it has been recognized as one of the most dangerous greenhouse gases, which has a direct effect on the global temperature rise [10]. Moreover, CO_2_ conversion into urea expands the stage of reactivity of this molecule under electrochemical conditions compared to the other well‐known and deeply studied CO_2_ reduction reaction (CO_2_RR) products [11]. Nitrogen‐containing precursors can be very different, ranging from nitrates (NO_3_^−^), nitrites (NO_2_^−^), nitrous oxides (NO_*x*_), and dinitrogen (N_2_). The use of nitrates has attracted more attention in the last few years because nitrates are well‐known pollutants, which are causes of eutrophication for water‐based ecosystems [12] and gastric cancer for humans [13]. The choice of nitrates is also dictated by their reactivity. The NO_3_^−^ nitrogen–oxygen double bond (N=O) dissociation energy is 204 kJ mol^−1^. This is more than 4 times lower compared to the nitrogen–nitrogen triple bond (N≡N) dissociation energy (941 kJ mol^−1^) in N_2_. In fact, molecular nitrogen, despite its abundance in air, requires high energetic inputs to be activated. Consequently, carbon dioxide and nitrates seem to be the most promising couple of reactants for urea electrosynthesis.

The development of urea electrosynthesis relies on different aspects. First, low‐cost and easy‐to‐make electrocatalysts should be pursued, which at the same time should be highly productive and selective for urea electrosynthesis. However, the electrocatalyst is only a part of the whole process. Fundamental insight should be devoted to selecting an engineered electrolyte to boost reaction efficiency. Also, electrochemical synthetic methodologies (constant current, constant potential, pulsed techniques, for example) and implementation of industrially relevant electrochemical cells (flow cells and membrane electrode assembly (MEA)) deserve attention.

**FIGURE 1:**
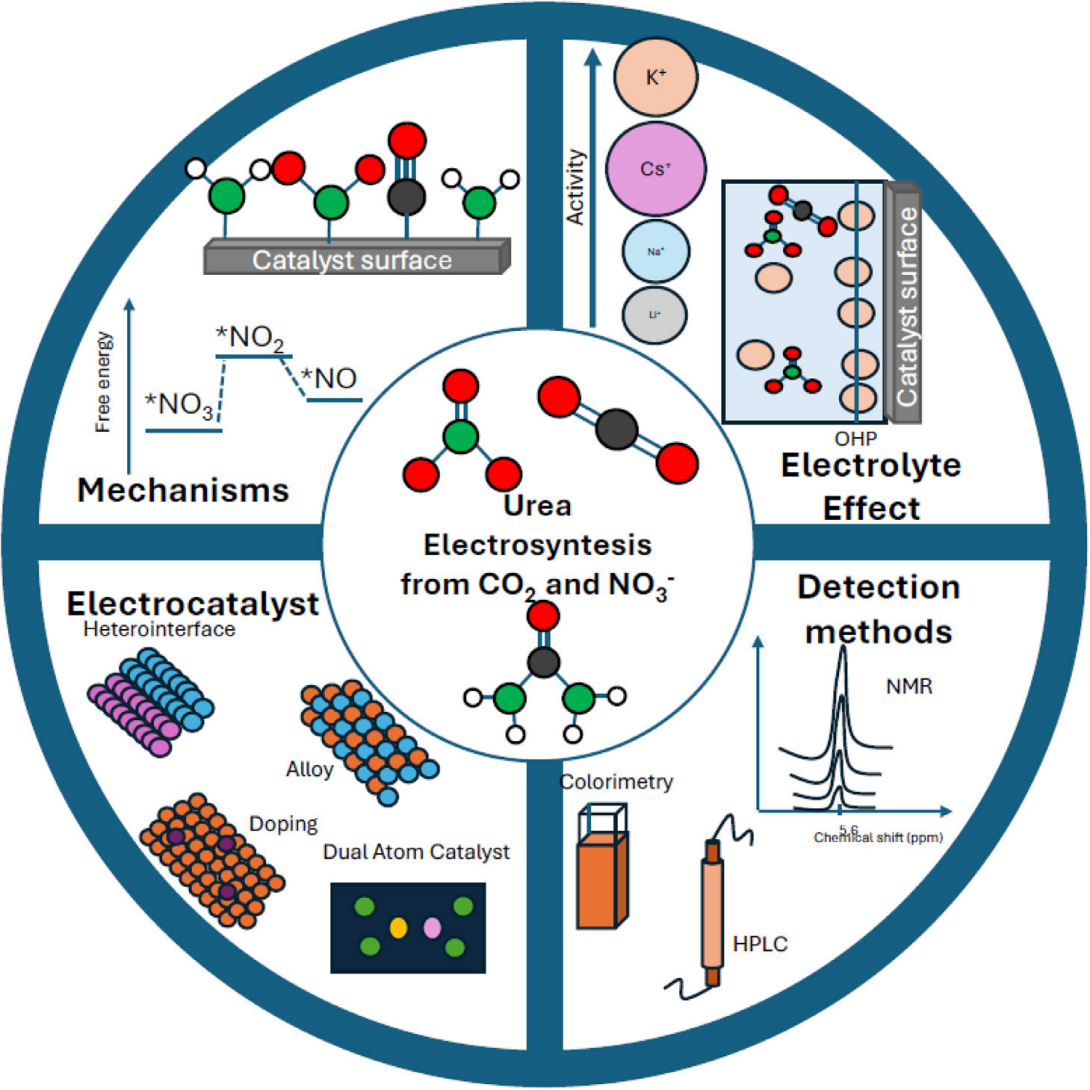
Schematic diagram depicting the four main sections of the present review paper.

Despite being an extensively researched topic, the technology of urea electrosynthesis still remains in its initial stage where the challenges prevail in synthesis, characterization, and benchmarking of the efficacious electrocatalysts. This review aims to collect the most recent advancements in the field of urea electrosynthesis from carbon dioxide and nitrates. A particular focus is devoted to the very last year (2025), concentrating effort on: 1) the advancement in reaction mechanism understanding, 2) the electrolyte effects, 3) the improved electrocatalysts, and 4) the urea detection methods in the electrocatalytic systems (Figure 1).

The text has been structured to provide meaningful yet comprehensive knowledge in the field of urea electrosynthesis. Through the four sections, readers will gain an understanding of the most fundamental techniques for the characterization of urea electrosynthesis mechanisms, encompassing both experimental and theoretical approaches, whose fundamental basis is generally not reported. Indeed, these bases need a careful contextualization for electrochemical systems. Moreover, a compilation of mechanisms proposed to date will offer a solid basis for the design of new atomic‐level electrocatalysts and optimized electrolyte composition to boost urea production performance. Urea detection and quantification are not trivial due to its low concentration and the presence of diverse species that can affect the analytical detection and quantification. There is a general consensus on misleading measurements for detecting and quantifying urea, with little consideration of the species that might cause false positives or enhance the quantification of urea. Therefore, the most recent methods for urea quantification are discussed, highlighting and deeply discussing their respective advantages and limitations. Overall, this review presents the latest scientific literature on urea electrosynthesis from carbon dioxide and nitrates and identifies the bottlenecks related to the topic. Finally, interesting perspectives and suggestions are given, highlighting possible solutions to commonly encountered issues in many aspects of the field of urea electrosynthesis.

## 2 Reaction Mechanisms

The development of new and efficient catalysts for urea electrosynthesis requires a renewed perspective at the atomic level. An essential first step toward understanding of this reaction lies in an accurate description of the fundamental mechanisms governing the formation of the key intermediates involved in urea electroproduction. A detailed, atomistic insight into which intermediates are preferentially generated on the electrocatalyst surface on a specific active site is therefore crucial for guiding the rational design of next‐generation electrocatalytic materials. Equally important is the understanding of the experimental and theoretical tools that enable the elucidation of these intrinsic mechanisms.

The half reactions in acidic and alkaline conditions for the urea electrosynthesis from CO_2_ and NO_3_^−^ are reported in reactions (1) and (2) [14]. The electrochemical conversion of carbon dioxide and nitrates into urea involves 16 electron transfer (ET) steps and 2 C—N coupling steps.

(1) $${\mathrm{CO}}_{2}+2{\mathrm{NO}}_{3}^{-}+18H^{+}+16e^{-}\;\to \;\mathrm{CO}{\left({\mathrm{NH}}_{2}\right)}_{2}+7H_{2}O\;\;\;E{}^{\circ}=+0.85V\;\mathrm{vs}\;\mathrm{SHE}$$

(2) $${\mathrm{CO}}_{2}+2{\mathrm{NO}}_{3}^{-}+11H_{2}O+16e^{-}\;\to \;\mathrm{CO}{\left({\mathrm{NH}}_{2}\right)}_{2}+18{\mathrm{OH}}^{-}\;\;\;E{}^{\circ}=-0.08V\;\mathrm{vs}\;\mathrm{SHE}$$

Although urea electrosynthesis is often described as a simultaneous reduction of CO_2_ and NO_3_^−^, this interpretation is only partially correct. The oxidation state of carbon remains +4 in both CO_2_ and urea, while nitrogen is reduced from +5 in nitrate to –3 in urea. So, from a formal point of view, the 16 electrons are used to reduce nitrogen, while the redox state of carbon remains unchanged. From this side, the urea electrosynthesis should not be called an alternative CO_2_RR approach but, more generally, a CO_2_ valorization methodology. Anyway, the underlying urea electrosynthesis mechanism involves CO_2_RR steps, even if at the end of the reaction, the electrons used to reduce CO_2_ are (formally) located on the nitrogen atoms. The reaction mechanisms involve several intermediates, which are generated after the adsorption of the reactants and following ET or proton/electron transfers (PET).

Also, C—N coupled intermediates are formed, which are essential for urea production. It is normally assumed that most of these intermediates are always adsorbed on the electrocatalyst surface. However, some of these can also be desorbed and subsequently react. Common intermediates are shared among the urea electrosynthesis, the CO_2_RR and the NO_3_^−^ reduction reaction (NO_3_RR) except for the C—N coupled intermediates. It is seldom reported that the two C—N coupling steps, crucial for urea formation, are chemical processes. No ET from the electrode is involved, but a pure chemical formation of a C—N covalent bond. From this perspective, the electrochemical potential (usually expressed in V vs RHE) dependence of urea electrosynthesis in terms of selectivity (or Faradaic efficiency (FE)) and yield (urea yield (UY) rate or partial current density *j*_urea_), which usually has a volcano plot‐like trend, should not be directly imputed to a more efficient electrochemical potential‐driven C—N coupling but to another electrochemical potential‐dependent parameter, such as adsorption energies and surface coverage, which ultimately impact reaction kinetics [15, 16, 17, 18, 19]. Because of its intrinsic chemical nature, the C—N coupling step could be favored by other factors. The spatial confinement and tandem or dual catalysis are considered promising strategies for efficient urea electrosynthesis and will be briefly described in the electrocatalytic materials section. The following two sections will focus on the most accredited mechanistic pathways for electrochemical urea synthesis and report advanced techniques for intermediates identification in operando conditions.

### 2.1 Reaction Mechanism Pathways

Multiple reaction pathways have been presented in the literature, based on both the experimental observation of some key intermediates and density functional theory (DFT) calculations. A first level of categorization differentiates the C—N coupling mechanisms according to the two fundamental mechanisms of heterogeneous catalysis: the Langmuir–Hinshelwood and the Eley‐Rideal mechanisms [7, 20].

In the Langmuir–Hinshelwood, the C‐ and N‐intermediates couple when they are still adsorbed on the electrode surface (*C + *N → *CN or CN*, where * denotes the atom involved in the adsorption).

In the Eley–Rideal mechanism, the C—N bond is formed when an adsorbed species reacts with a dissolved intermediate, usually produced in a preceding step and further desorbed (C + *N → CN* or *C + N → *CN). A deeper level of description has been recently reported by Zhou et al., who have reviewed 13 different reaction pathways during urea electrosynthesis based on the nature of the intermediates involved in the C—N coupling. These pathways are then categorized into nine structural flow patterns based on different C—N coupling modes. Ultimately, the flow patterns are divided into four types: coupling of *CO and *NH_2_; coupling of *CO and *NO; coupling of *CO and other N‐containing intermediates; coupling of CO_2_ and N‐containing intermediates [21]. Table 1 reports all 13 reaction sequences.

**TABLE 1:** The 13 reaction sequences for urea electrosynthesis developed up to 2025. This table was readapted from [21] with permission, Copyright 2025, Wiley.

Types	Structural flow pattern	Reaction pathways
1	*NO_3_ → *NH_2_ → *CONH_2_ → urea	*CO_2_ → *CO I, *NO_3_ → *NHO → *NHOH → *NH → *NH_2_ → urea II, *NO_3_ → *NHO → *NHOH → *NH_2_OH → *NH_2_ → urea III, *NO_3_ → *NHO → *N → *NH → *NH_2_ → urea
2	*NO_3_ → *NO → *ONCO → urea	*CO_2_ → *CO IV, *NO_3_ → *NO → *ONCO → *ONCONO → *NCONOH → urea V, *NO_3_ → *NO → *ONCO → *ONCONO → *ONCONH → urea VI, *NO_3_ → *NO → *ONCO → *ONCONO → *HONCON → urea
3	*NO_3_ → *NO_2_ → *CONO_2_ → urea *NO_3_ → *NOOH → *CONOOH → urea *NO_3_ → *NOH → *CONOH → urea *NO_3_ → *NH → *NHCO → urea	*CO_2_ → *CO VII, *NO_3_ → *NO_2_ → *CONO_2_ → *NO_2_CONH_2_ → urea VIII, *NO_3_ → *NOOH → *CONOOH → *NCONOOH → urea IX, *NO_3_ → *NOH → *CONOH → *NCONOH → urea X, *NO_3_ → *NH → *NHCO → *NHCONO → urea
4	*NO_2_ → *NO_2_ → *CO_2_NO_2_ → urea *NO_3_ → NO_2_^‐^ → *CO_2_NO_2_ → urea *NO_3_ → *NH → *NHCO_2_ → urea	XI, *NO_3_ → *NO_2_ → *CO_2_NO_2_ → *NO_2_CONH_2_ → urea XII, *NO_3_ → NO_2_^−^ → *CO_2_NO_2_ → *NHCO_2_ → urea XIII, *NO_3_ → *NH → *NHCO_2_ → *NHCONH_2_ → urea

Zhou et al. have listed the reaction mechanisms occurring during urea electrosynthesis according to the first C—N coupling step in four categories (*NO_2_ + *CO_2_; *NO_2_ + *CO; *NO  + *CO; and *NH_2_ + *CO) [22]. The Langmuir–Hinshelwood mechanism is widely accepted for the C—N coupling steps on a very wide range of urea electroactive materials. Commonly, it involves two subsequent C—N couplings between *CO and *NH_2_ to achieve urea [23, 24, 25]. However, less reduced species than *CO and *NH_2_ can undergo the C—N coupling, and it is called “early C—N coupling.” Li et al. have identified an early C—N between *CO_2_ and *NH_2_ into *OCONH_2_ by in situ attenuated total reflectance surface‐enhanced infrared spectroscopy (ATR‐SEIRAS) on carbon quantum dot/copper metal–organic framework (MOF) heterostructure (Cu‐MOF‐CQD) and it is supported by DFT calculations [26].

Liu et al. have reported an early C—N coupling on amorphous indium–boron oxide (A‐In@BO_*x*_) between *COOH and *NO_2_, noting that when CO is used as a feeding gas instead of CO_2_, no urea is produced [27]. Cheng et al. have reported two subsequent early C—N couplings between *CO and *NO on amorphous CuO_*x*_‐coated crystalline Cu nanowires (C‐Cu‐A_4_), supported by DFT calculations. In the same work, electron paramagnetic resonance (EPR) spectroscopy, X‐ray absorption spectroscopy (XAS), and Auger electron spectroscopy (AES) have demonstrated an unusual electrochemical–chemical looping mechanism for the generation of *NO_2_ from NO_3_^−^. Indeed, under the electrochemically relevant conditions for urea electrosynthesis, Cu^0^ and oxygen vacancies (O_v_) are generated on the surface; then, O_v_ mediates NO_3_^−^ activation via O‐atom insertion, yielding *NO_2_ while Cu^0^ is oxidized to Cu^+^. This pathway avoids the initial 2 ET NO_3_^−^ reduction, which is usually the rate‐determining step for NO_3_RR [28], reducing the electrons required for urea electrosynthesis from 16 to 12 [29].

By DFT calculations, Feng et al. have corroborated another early C—N coupling mechanism on an atomic‐scale Mott–Shottky analogy in a Cu–Sn alloy (Sn_2_Cu). The first C—N coupling involves *CO and *NHO, for which the protonation energy barrier to *NHOH is not favored on this material, while the second C—N coupling proceeds between *CONH_2_ and *NO [30]. The Eley–Rideal mechanism is rarely reported in the literature, probably due to difficulties of verification. For example, attenuated total reflection Fourier‐transform infrared (ATR‐FTIR) spectroscopy and related techniques, commonly used for intermediate identification, are not sensitive to distinguish between adsorbate and desorbed species, which are still close to the electrode surface, at least in the range of 1 µm [31]. In an interesting mechanistic study, Hu et al. have shown that the first C—N coupling takes place between *NO_2_ and free CO_2_ on a polycrystalline copper electrode, if the potential is not driven so negatively to activate CO_2_RR [32]. Chen et al. have reported a probable Eley–Rideal mechanism between desorbed NH_2_OH and *CO on Ga–Y dual‐atom catalyst (DAC), supported by DFT and in situ ATR‐SEIRAS [33]. Zhai et al. have suggested an early C—N coupling between CO_2_ and *NO_2_ on a α‐CuZn alloy [34].

### 2.2 Toolkit to Study the Urea Electrosynthesis Reaction Mechanisms

In situ and operando techniques have been extensively developed to elucidate the reaction mechanisms of both CO_2_RR and NO_3_RR, and they can likewise be applied to the coelectrolysis of CO_2_ and NO_3_^−^ toward urea. Here, three widely used operando techniques relevant to studying the reaction mechanism are briefly discussed, namely, ATR‐FTIR, differential electrochemical mass spectrometry (DEMS), and EPR. In addition, DFT calculations are highlighted due to their widespread use in electrocatalysis, with particular emphasis on key considerations that help readers critically interpret computational results in the literature.

Operando ATR‐FTIR spectroscopy finds extensive use in electrochemistry to investigate surface and near‐surface phenomena [31]. It basically measures the characteristic vibrational features of adsorbates and of those species close to the electrode. In a normal three‐electrode measurement, the working electrode (with the electroactive material deposited and pressed on an IR‐transparent crystal), the counter electrode, and the reference electrode are immersed in the electrolyte and then in a suitable spectroelectrochemical cell.

In this case, the IR beam is directed from the crystal side and only an evanescent wave, whose penetration depth is in the order of a few micrometers, is transmitted to the sample. After multiple reflection/transmission steps, the attenuated beam reaches the detector. The experiment is normally conducted starting from the open‐circuit potential (OCP) and approaching more negative potential. The potential profile is characterized by a step size (usually in the order of 0.1 V) and a potentiostatic time (in the order of a few minutes). Quantitative evaluations are normally done by tracking the abundance of a given intermediate, identified by its characteristic wavenumber, from its FTIR intensity evolution. However, in the context of electrocatalysis, the electrode local environment is highly dependent on the reaction‐specific rate (the current density). Consequently, intensity trends could be falsified by local phenomena related, for example, to limited mass transport or pH alteration and not to the coverage solely [35, 36, 37]. These kinds of spectroscopic–kinetic correlations have not been addressed for the urea electrosynthesis. Anyway, ATR‐FTIR has been widely applied for the detection of the most abundant intermediates, especially in its variation named ATR‐SEIRAS, which allows for an increase in the signal‐to‐noise ratio [31]. The most common intermediates are listed in Table 2 and can be used as a reference in a first approximation. These data were collected from recent available literature [26, 38, 39, 40, 41, 42, 43, 44, 45, 46]. In parallel, recent reviews by Zhu et al., Yin and Wang and Fu et al. contain characteristic wavenumbers of several CO_2_RR and NO_3_RR intermediates [47, 48, 49].

**TABLE 2:** Characteristic wavenumber of the most common intermediates or bonds occurring during urea electrosynthesis.

Bond/Intermediate	**Wavenumber, cm** ^**−1**^	Reference
C—N	1410, 1412, 1417, 1435, 1450, 1460, 1475	[26, 35, 36, 37, 38, 39, 40, 41]
N—C—N	1305, 1455, 1488	[26, 42]
CO_2_NH_2_	1390, 1542, 1635	[26, 35]
COOHNH_2_	1690, 1710	[35, 43]
CONH_2_	1637, 1650, 1720	[36, 41, 42]
OCNO	2050, 2077	[38, 39]
CO_2_NO_2_	1447	[43]
NHCO	1698	[40]
NH_2_ urea	1099, 1180, 1184, 1300, 1302, 1600, 1641	[36, 37, 43]
C=O urea	1662, 1647, 1737	[37, 39, 40]

DEMS is an analytical technique that enables real‐time detection and quantification of volatile and gaseous species produced or consumed at the electrode surface during an electrochemical reaction [44]. DEMS operations are based on the direct coupling of an electrochemical cell with a mass spectrometer through a selective, gas‐permeable interface, typically a hydrophobic membrane. This membrane allows reaction‐generated molecules to pass into the vacuum system of the mass spectrometer while preventing the liquid electrolyte from entering. During operation, electrochemical processes are initiated at the working electrode, and any volatile products formed diffuse through the membrane into the mass spectrometer. The instrument then ionizes these species and separates them according to their mass‐to‐charge ratio, enabling rapid and sensitive detection. Because DEMS provides time‐resolved measurements, it allows researchers to correlate electrochemical signals, such as potential steps or current variations, with the simultaneous formation or consumption of specific molecules. From this point of view, DEMS could be more sensitive to the abovementioned local phenomena since almost direct time correlation between the current and intermediates is tracked. Moreover, because it exploits a mass detector, DEMS can also differentiate isotopes. In the field of urea electrosynthesis, the typical implementation of DEMS consists of stepping the potential from OCP to a given cathodic potential, identifying intermediates that are both stable in the gas phase (CO, NH_3_ and NO_*x*_) but also which are normally only present as adsorbed species (*COOH and various C—N coupled species). Niu et al. have conducted a DEMS experiment by stepping the potential multiple times from OCP to −0.7 V versus RHE on W‐doped CuO and have identified CO_2_NO_2_, CO_2_NH_2_, and COOHNH_2_. So, they have supposed an early C—N coupling between *CO_2_ and *NO_2_ [43]. Isotopic experiments conducted in the presence of ^15^NO_3_^−^ or ^13^CO_2_ are easily conducted with an online DEMS to verify the N‐ and C‐origin. Basically, it is required to check if some selected (*m*/*z*) signals are observable in the corresponding (*m*/*z*) + 1 state after isotopic electrolyte labeling [45, 46]. An interesting DEMS mechanistic investigation has been proposed by Zhang et al., which has tracked urea, NO_2_, NO, NH_4_^+^, and CH_4_ signal by pulsing the potential between −0.5 and −0.7 V versus RHE. CH_4_ has been used as a marker of the C‐pathway and its signal intensifies at −0.5 V, while NO, a marker of the N‐pathway, is predominant at −0.7 V. This behavior correlates with the dynamic length of the Cu—O bond in the electrocatalyst (Cu_5_Cl_2_(μ_3_‐OH)_2_ porous honeycomb‐like 3D framework), which is longer at −0.5 V and shorter at −0.7 V [47].

Quasi‐in situ EPR is an analytical approach designed to capture (or trap) and characterize paramagnetic intermediates formed during electrocatalytic reactions [48]. This strategy allows the preservation of short‐lived or reactive paramagnetic species that would otherwise decay before analysis. In a typical quasi (or semi) in situ experiment, the electrocatalyst, often supported on a conductive substrate or deposited as a thin film, is first polarized at a defined potential inside a dedicated electrochemical cell. Immediately after the specific intermediate is generated, this is captured by a trapping agent, forming an EPR‐active molecule.

The EPR measurement is then carried out to detect changes in spin state, *g*‐value, and hyperfine structure associated with the electrochemically generated species. An interesting application of quasi‐in situ EPR is related to the study of water dissociation on the electrocatalyst's surface (*H_2_O → *H + *OH) during urea electrosynthesis. The water dissociation reaction is usually cited as a key but indirect step that can hamper urea selectivity and productivity. Indeed, the urea electrosynthesis consumes protons and those materials that can easily regenerate protons seem to be good candidates for urea electrosynthesis. The typical quasi‐in situ EPR experiment for *H analysis makes use of dimethyl‐1‐pyrrolidine‐N‐oxide (DMPO), which is a commonly used radical scavenger. When a suitable potential is applied to a *H‐generating material, its surface becomes partly covered by *H. These species react with DMPO and become DMPOH, which is EPR‐active. DMPOH has a characteristic EPR signal (*g* around 2.005–2.006) and hyperfine structure relative intensities 1:1:2:1:2:1:2:1:1 [49].

**FIGURE 2:**
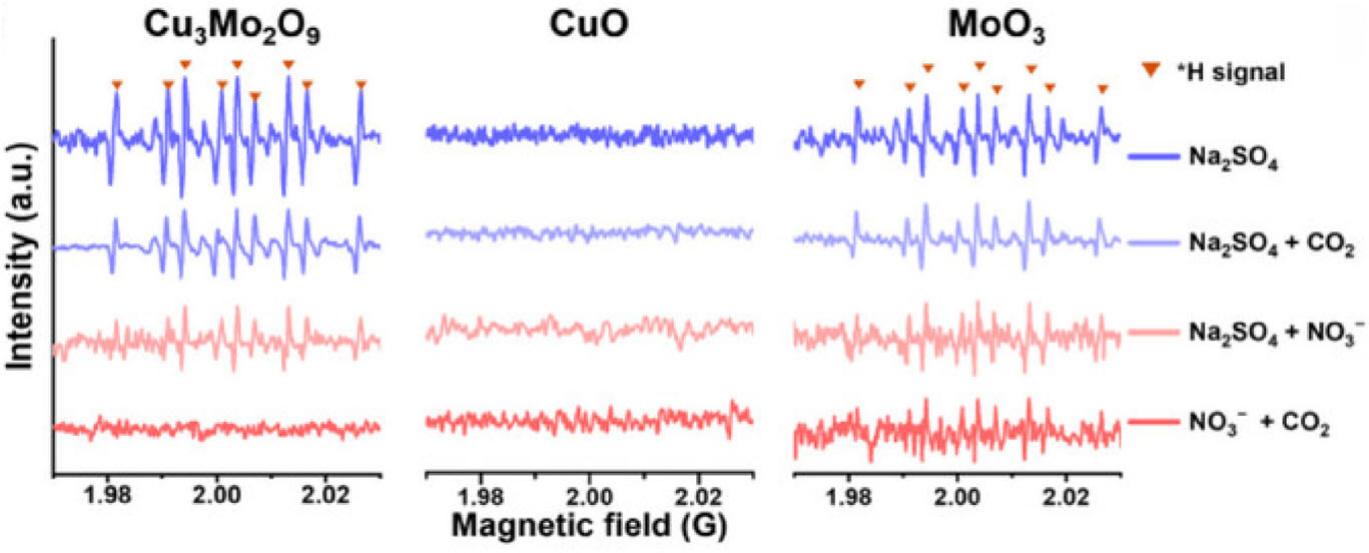
Quasi‐in situ EPR spectra of the solutions obtained after 10 min of electrolysis by Cu_3_Mo_2_O_9_, CuO, and MoO_3_ at −0.8 V versus RHE using DMPO as the *H‐trapping reagent. This image was taken from [50] with permission, Copyright 2025, Wiley.

An example of this technique has been proposed by Zhang et al., who have conducted quasi‐in situ EPR in the presence of DMPO on three different materials: CuO, MoO_3_, and Cu_3_Mo_2_O_9_. CuO did not show any DMPOH signal, both in CO_2_ sat. 0.1 M Na_2_SO_4_ or in CO_2_ sat 0.1 M Na_2_SO_4_ + 0.1 M KNO_3_, indicating that CuO fails in producing *H. MoO_3_ has similar and quite intense DMPOH signals in both electrolytes, pointing to the fact that this material is only able to evolve hydrogen (HER). Lastly, Cu_3_Mo_2_O_9_ shows a similar DMPOH signal compared to MoO_3_ in NO_3_^−^‐free electrolyte, but no signal is registered when CO_2_ is present. These trends clarify that Mo helps to dissociate water, but it is the synergy with Cu that determines *H consumption in the urea electrosynthesis rather than the HER (Figure 2) [50]. Oxygen vacancies (O_v_) can also be tracked by EPR because of their paramagnetic nature [51]. Cheng et al. have used EPR spectroscopy to observe the O_v_ filling mechanism mediated by nitrate, which then directly converts into *NO_2_, observed by a diminished EPR O_v_ signal intensity [29].

**FIGURE 3:**
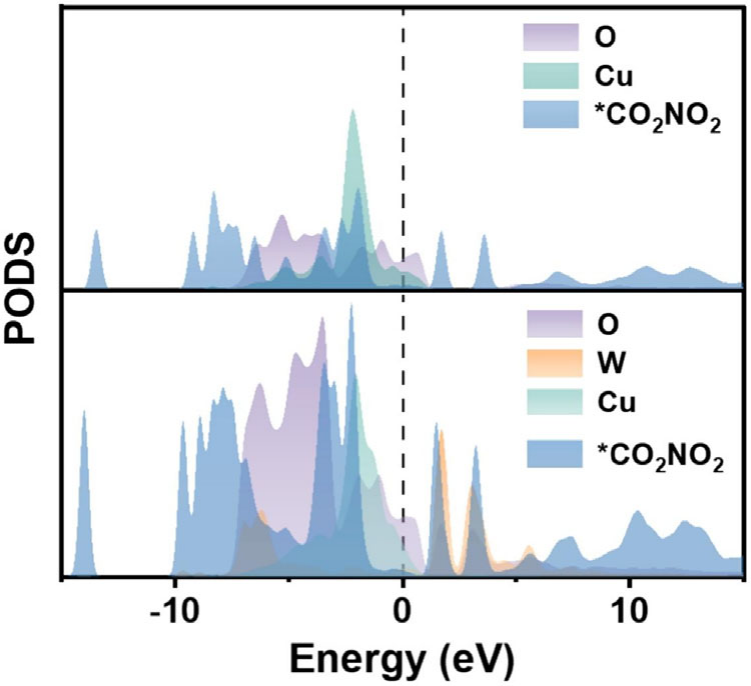
PDOS profiles of adsorbed *CO_2_NO_2_ on CuO and W‐doped CuO surfaces. This image was taken from [43] with permission, Copyright 2025, Elsevier.

DFT calculations are also a useful tool for the identification of reaction pathways in the context of electrocatalytic reactions [52]. Briefly, three commonly reported theoretical tools are the projected density of states (PDOS), the crystal orbital Hamiltonian population (COHP), and free energy diagrams. The PDOS is a representation of how electronic states are distributed in a material, resolved by specific atoms or orbitals. While the total density of states shows how many electronic states exist at each energy, the PDOS reveals which atoms or orbitals contribute to those states. This makes it a valuable tool for understanding the electronic structure of catalytic surfaces. In electrocatalysis, the PDOS is widely used to identify the nature of active sites and to analyze how catalysts interact with reaction intermediates. By examining the states near the Fermi level, it is possible to determine which orbitals participate in bonding during the reactions. Overlap between the PDOS of the electrocatalyst and that of an adsorbate indicates hybridization and provides insight into adsorption strength. For transition metals, the PDOS of the *d*‐orbitals is also used to determine the *d*‐band center, a key descriptor that correlates with the electrocatalytic activity. Overall, the PDOS helps to explain how modifications such as alloying, doping, and other engineering methodologies influence the electronic properties and therefore the performance of electrocatalysts. For example, Xing et al. have calculated the PDOS profile of Ru and Bi in Ru–Bi single atoms anchored on amorphous MnO_2_, which suggests strong *d*(Ru)–*p*(Bi) hybridization. Then, the comparison of the Ru PDOS with *CONH_2_ PDOS has revealed that only Ru is responsible for this key intermediate adsorption [53]. Also, Niu et al. have found a strong hybridization between 5d(W) and *CO_2_NO_2_ antibonding states near the Fermi level on W‐doped CuO (Figure 3) [43].

Crystal orbital Hamilton population (COHP) analysis is a valuable tool for resolving the energetic nature of interatomic interactions in solid‐state electrocatalysts. By decomposing the electronic structure into bonding (negative COHP) and antibonding contributions (positive COHP), COHP analysis enables identification of electronic states that stabilize or weaken the active site. The corresponding integrated COHP (ICOHP) provides a quantitative measure of bond strength, allowing direct comparison of metal–adsorbate, metal–ligand, or lattice–oxygen interactions central to catalytic performance. In electrocatalysis, stronger (more negative) ICOHP values typically correlate with optimized adsorption energetics, whereas significant antibonding occupation near the Fermi level may indicate potential instability or facile intermediate desorption. For example, Zhai et al. have calculated the COHP for Cu—O and Zn—O bonds in α‐CuZn and β‐CuZn in the presence of *NO_2_. The ICOHP was −0.719 eV for the Cu—O bond, much lower than −0.443 eV for Zn—O on α‐CuZn, indicating that *NO_2_ interacts more strongly with the Cu site in this phase. On the other side, on β‐CuZn, the ICOHP for Cu—O (−0.446 eV) and Zn—O (−0.449 eV) are similar. These factors correlate with better *CO_2_ insertion and C—N coupling on α‐CuZn [34]. Chen et al. have calculated the COHP for a PdAuCuIrCo high‐entropy alloy (HEA) and a AuCuIrCo medium‐entropy intermetallic (MEI) compound in the presence of *CO_2_ and *NO_2_. The ICOHP values serve as a measure of the C—N coupling interaction, with more negative ICOHP values indicating stronger C—N coupling capabilities. The HEA has an ICOHP of −7.46, higher than −6.46 observed for the MEI, suggesting the HEA is a more effective catalyst for C—N coupling than the MEI, in line with electrochemical tests [54].

Free energy diagrams are typically constructed using the computational hydrogen electrode (CHE) approach, introduced by Nørskov et al. in 2004, which provides a consistent framework for modeling PET reactions within DFT [55]. In this method, the chemical potential of a proton–electron pair (H^+^ +e) is directly referenced to half a hydrogen molecule (1/2 H_2_) at 0 V RHE, effectively allowing the treatment of electrochemical steps without explicitly including solvated protons or electrons. Indeed, the chemical potential of ½ H_2_ can be easily calculated by DFT means. In the CHE framework, the free energy of each electrochemical intermediate is calculated by combining DFT‐derived electronic energies with thermodynamic corrections and a potential‐dependent term. Concretely, for an adsorbed species *X on the model electrocatalyst surface, one first performs a DFT calculation to obtain its total electronic energy, *E*_elec_(*X). Then, zero‐point energy (ZPE) corrections (enthalpic term), finite‐temperature contributions (entropic term), and a bias term (applied potential contribution) are added to get the Gibbs free energy, as reported in Equation (3). Eventually, a pH‐correction term can be added to avoid the pH = 0 restriction of the SHE.

(3) $$G\left(X^{*}\right)=E_{\mathrm{elec}}\left(X^{*}\right)+\Delta E_{\mathrm{ZPE}}-T\Delta S-neU(+k_{B}Tln10\cdot pH)$$

Δ*E*_ZPE_ is derived from vibrational frequency analysis, Δ*S* accounts for vibrational (and, if relevant, translational/rotational) entropy, *T* is the absolute temperature, *n* is the number of electrons involved in the ET, *e* is the elementary charge, and *U* is the applied potential versus SHE. Then, the free energy diagram is plotted considering all the relevant intermediates. Typically, the largest thermodynamic overpotential (usually positive) between two reaction intermediates is taken as the potential‐limiting (or ‐determining) step (PDS) of the overall reaction. The PDS is also usually assigned as the rate‐determining step (RDS). Although PDS and RDS may coincide, their definitions are fundamentally different because the PDS has a thermodynamic nature (the step with the more positive free energy change) while the RDS has a kinetic meaning (the step with the highest activation barrier). Since activation barriers and transition states are not calculated in the CHE method, PDS should always be used for the higest endergonic steps. For a better insight and understanding of the theory, Koper's papers on multi‐ET reactions are strongly suggested [56, 57]. Anyway, as a result, many studies conclude that one material outperforms another solely because it exhibits a lower thermodynamic overpotential. This practice is especially common within the urea electrosynthesis field, where the CHE method is widely used due to its low computational cost. For example, Deng et al. have recognized that the *CO_2_ to *COOH step was more favorable on P‐doped Cu/Fe_2_O_3_ heterojunction than the nondoped material because of the lower thermodynamic barrier (0.96 vs. 1.04 eV) [58]. Xu et al. have compared pristine In_2_O_3_ and Cu nanoparticle‐supported In_2_O_3_, assessing PDSs were the *NO to *NHO hydrogenation (0.67 eV) and the *NH_2_O to *NH_2_OH hydrogenation (0.36 eV) in the NO_3_RR side. In the CO_2_RR side, Cu–In_2_O_3_ has been calculated to be more performing than In_2_O_3_ for the *COOH to *CO step (0.96 vs. 3.05 eV) [59]. Zhai et al. have found that the C—N coupling between *CO_2_ and *NO_2_ is more favorable on α‐CuZn (Δ*E* = 1.10 eV) compared to β‐CuZn (ΔE = 1.26 eV) [34]. However, as extensively discussed by Exner, the CHE approach is inherently insufficient for assessing electrocatalytic activity [60]. The thermodynamic overpotential alone is a fundamentally limited descriptor: the CHE framework provides neither kinetic information nor a realistic description of the actual overpotential. Indeed, activation barriers are not calculated and the *‐neU* correction in Equation (3) assumes that the composition and structure of the electrocatalyst remain unchanged by increasing the driving force. Therefore, Exner proposed the use of alternative descriptors, such as ESSI (Electrochemical Step Symmetry Index) and *G*_max_(η) (free‐energy span to move from the reaction intermediate with the smallest free energy to the reaction intermediate with the highest free energy in the free‐energy landscape at a given overpotential value) [60], which unfortunately have not yet been implemented in urea electrosynthesis theoretical studies. Conclusively, it is recommended that free‐energy diagrams should be used with caution within the urea electrosynthesis context. Relying solely on the thermodynamic overpotential as an activity descriptor can be misleading, and more extended evaluations are required.

In summary, a basic yet comprehensive understanding of the mechanisms underlying urea electrosynthesis, together with the experimental and theoretical techniques employed to probe them, has been presented here. This understanding represents a critical step toward advancing this field since the rational design of new electrocatalysts and the stabilization of critical intermediates are deeply interconnected. By integrating mechanistic insights with state‐of‐the‐art characterization methods and computational approaches, it becomes possible to identify the key intermediates and rate‐limiting steps that govern the reaction pathway. Such knowledge not only deepens our fundamental understanding but also provides a solid foundation for the rational design of more efficient and selective catalytic systems.

## 3 Electrolyte Effects

The mechanistic pathways discussed above are intrinsically linked to the properties of the electrolyte, which plays a multifaceted role in electrocatalytic systems. Beyond serving as a medium for ion transport, the electrolyte can directly influence the stabilization of reaction intermediates, the local reaction environment, and the availability of key reactants at the electrocatalyst surface. Variations in pH, ionic strength, and the nature of the electrolyte ions can alter adsorption energies, modulate interfacial electric fields, and ultimately shift reaction pathways and selectivity. A comprehensive understanding of urea electrosynthesis mechanisms cannot be decoupled from the effects of the electrolyte, as the interplay between these factors is essential for accurately describing and optimizing catalytic performance. Moreover, the effect of the electrolyte has been rarely reviewed and summarized for the reaction of urea electrosynthesis. So, the present text represents a strong starting point for people facing this aspect for the first time.

**FIGURE 4:**
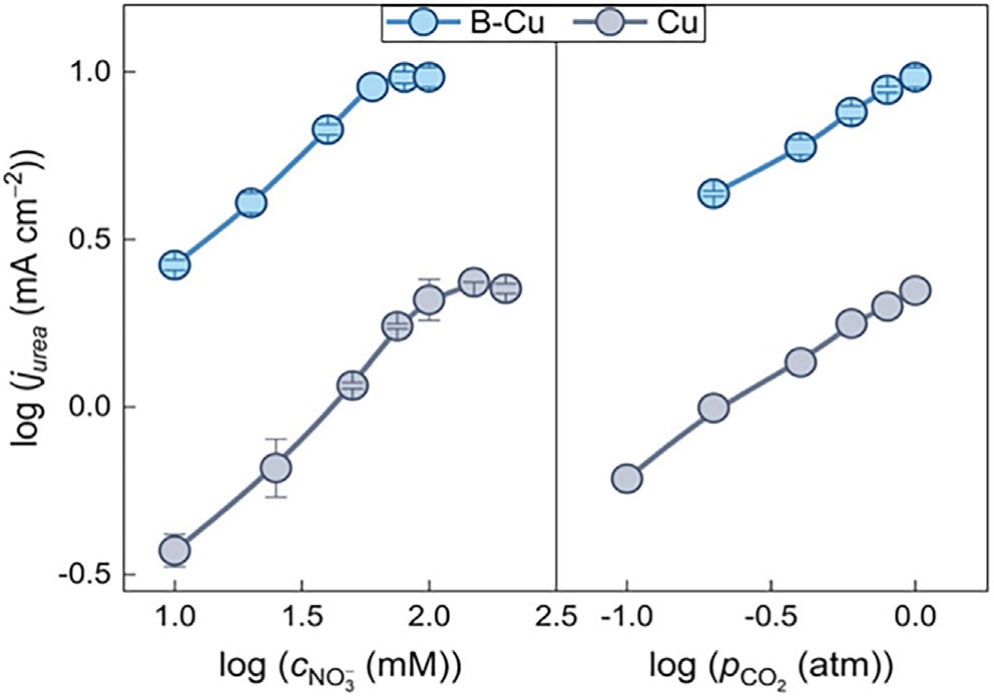
Urea partial current density dependence on NO_3_^−^ concentration and CO_2_ partial pressure on B–Cu and Cu at 0.05 V versus RHE. This image was taken from ref. [32] with permission, Copyright 2025, American Chemical Society.

Both a rapid survey and a thorough review of the existing literature—including works published before 2025—confirm that electrolyte effects have been only marginally explored in the context of urea electrosynthesis. Nevertheless, electrolyte effects have been shown to play a crucial role in determining the activity of various model electrocatalysts for HER, CO_2_RR, and NO_3_RR, and several mechanistic interpretations have been proposed based on experimental and theoretical studies. The term *“*electrolyte effect*”* encompasses multiple factors, among which pH, cation identity, anion identity, and electrolyte concentration are likely the most significant. As recently reviewed by Sebastiàn‐Pascual et al., three main interpretative frameworks have emerged to explain these effects: modulation of adsorption energies, variations in the electric field within the double layer, and the reorganization of the water structure [61]. However, the current understanding of electrolyte effects in urea electrosynthesis remains limited, and achieving a comparable level of theoretical insight is not feasible at present. Among the parameters mentioned above, electrolyte concentration holds particular importance. Indeed, CO_2_RR and NO_3_RR have different kinetics; NO_3_RR is faster than CO_2_RR because of CO_2_ high inherent stability and its limited mass transport [62]. It is well‐known that reaction rates are concentration‐dependent. Consequently, it is crucial to determine how the reactants’ concentrations affect both selectivity and yield in electrocatalytic reactions. Unfortunately, many papers do not report the “absolute” maximum selectivity and yield for a given electrocatalyst/electrolyte couple. Indeed, the urea electrosynthesis is commonly conducted using CO_2_‐saturated bicarbonate and nitrate aqueous electrolytes. The use of bicarbonate is a reminiscence of the CO_2_RR. Indeed, CO_2_ is few soluble in water (around 33 mM at 1 atm and room temperature [63, 64]) and bicarbonate can easily regenerate free CO_2_ close to the electrode through the acid–base equilibria between CO_2_ and bicarbonate themselves, preventing important mass transport limitations in static conditions. Because of this acid–base equilibrium, bicarbonate also has a buffering power, which, however, is quite limited since CO_2_ (the conjugate acid) is poorly concentrated [65, 66]. This could have potential effects on the urea electrosynthesis performance, especially related to pH effects, which will be discussed later. On the other hand, alkali metals’ nitrates are highly soluble in water and are not involved in any acid–base equilibria at neutral pH. A commonly used electrolyte to test the urea electrosynthesis activity of a newly prepared electrocatalyst is an equimolar nitrate–bicarbonate solution, usually at 0.1 M each. This choice is more simplistic than rationalized. Because NO_3_RR has an intrinsic higher kinetics, a higher nitrate concentration could favor nitrite or ammonia generation and saturate urea electrosynthesis active sites. Also, the reaction stoichiometry (2N:1C) is misleading for the adjustment of the electrolyte concentration. Hu et al. have investigated the C—N coupling mechanism on boron‐doped copper as model electrocatalyst by varying the CO_2_ partial pressure from 0.1 atm (3.3 mM) to 1.0 atm (33 mM) and nitrate concentration from 0.01 to 0.20 M. Urea electrosynthesis has been tested at 0.05 V versus RHE in 1 M KOH and showed linear increase across the CO_2_ concentration range while it plateaus as nitrate concentration approaches 0.06 M. These trends supported an Eley‐Rideal mechanism between CO_2_ and *NO_2_ but also simply show that there is an optimal nitrate concentration. On the other side, 1 atm of CO_2_ was demonstrated to be effectively the most efficient (Figure 4). Also, a wastewater‐like nitrate concentration (500 ppm) was tested to enhance urea selectivity to 60% [32].

**FIGURE 5:**
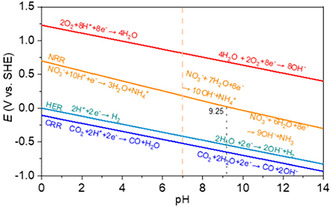
E‐pH plot for the CO_2_RR to CO, HER, NO_3_RR to NH_4_^+^, and OER. This image was taken from [62] with permission, Copyright 2026, Elsevier.

Song et al. have studied the nitrate concentration effect on Zn‐doped defect‐rich Fe_2_O_3_, finding that 0.1 M nitrate is the optimal concentration in the range from 0.01 to 0.2 M, while the CO_2_ partial pressure was always kept to 100% [67]. Li et al. have tested the urea electrosynthesis performance of oxidized carbon nanotubes in the presence of 1000 ppm NO_3_^−^ (16 mM), which is closer to nitrates downstream water concentration [68]. Yao et al. have conducted urea electrosynthesis in 0.1 M nitrate electrolyte bubbled with different CO_2_/O_2_ and CO_2_/N_2_ mixtures to simulate flue gases. They found that UY increased in the presence of O_2_ and N_2_ using BiVO_4_ incorporated in a MOF, despite no clear demonstration having been given for this behavior [39]. For what concerns the pH effect, in 2025, only Wu et al. have performed a systematic evaluation of urea electrosynthesis in 0.1 M KNO_3_ with pH ranging from 1 to 14 using a Cu–Sn dual‐atom electrocatalyst. They have revealed that the performance is highly suppressed at pH < 4 and pH > 9 [69]. Anyway, the CO_2_RR and NO_3_RR proton consumption makes urea electrosynthesis sensitive to pH (Figure 5) and more investigation should be devoted to the topic.

**FIGURE 6:**
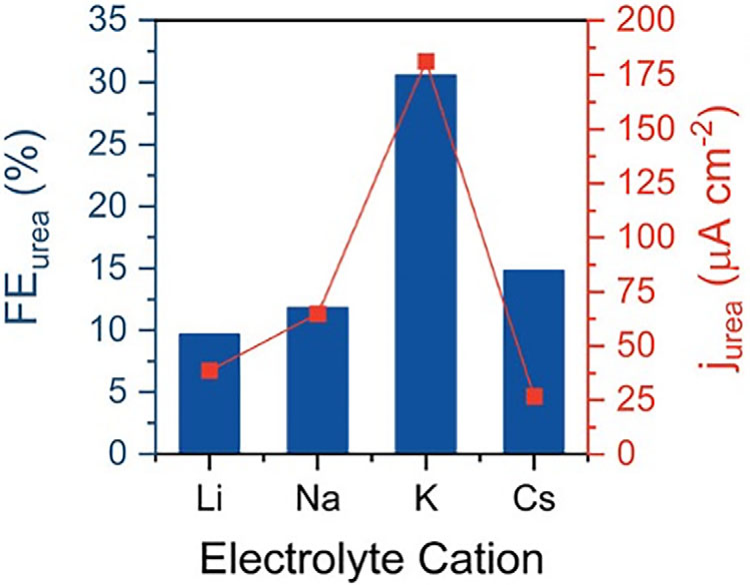
Urea selectivity and yield dependence on 0.1 M alkali metal cation identity at pH 3 and −0.3 V versus RHE. This image was taken from [73] with permission, Copyright 2023, American Chemical Society.

As recently reported by Ferri [70], a missing pH control could result in product distribution fluctuations during a classical potentiostatic or amperostatic experiment because of both local pH variations for low currents, but also of bulk pH variation during massive urea electroproduction. Ferri et al. have also suggested the implementation of alkaline electrolytes (KOH‐based) for urea electrosynthesis because of the inherent HER suppression ability. Anyway, the carbonate precipitation issue and urea instability in strongly alkaline conditions are limiting factors that should be addressed [70]. Despite anions’ effects seeming to have a minor role during cathodic reactions, particularly when the electrode is highly negatively polarized (or at least negatively shifted compared to the potential of zero charge) [71, 72], the identity of the cation has been largely studied in the context of CO_2_RR but also for the NO_3_RR [64, 72]. In 2023, Gerke et al. demonstrated that alkali cations influence urea selectivity and yield on Au electrodes, following the trend K^+^ > Cs^+^ > Na^+^ > Li^+^ (Figure 6) [73].

In 2024, Tu et al. also found this trend on single‐atom Co supported on TiO_2_ [74]. In the 2025 scenario, only a theoretical study combining DFT and AIMD (artificial intelligence molecular dynamics) has suggested the role of Bmim^+^ (1‐buthyl‐3‐methylimidazolium) at enhancing local CO_2_ concentration and ruled a NH_4_SO_4_ > BF_4_^− ^> K^+^ > NH_4_^+^ > Cu^2+^ trend for urea electrosynthesis on a CuAu single nanocrystal as model electrocatalyst.

Conclusively, we believe that the incoming years will be productive for intensive studies related to the electrolyte effects, both for mechanistic purposes and for practical engineering applications.

## 4 Electrocatalytic Materials for the Urea Electrosynthesis

Even considering the important role of the electrolyte, it becomes clear that the properties of the electroactive material ultimately govern the catalytic response of the system. While the electrolyte shapes the local reaction environment, it is the electrocatalyst surface that dictates the adsorption, activation, and transformation of reactants into desired products. The atomic structure, electronic properties, and surface composition of electroactive materials determine the binding strength of intermediates and the accessibility of reaction pathways. The electrolyte–electrocatalyst interaction remains important, but the design of advanced electroactive materials stands as the central point for controlling activity and selectivity. Therefore, a deeper focus on tailoring material properties is essential for driving progress in electrocatalysis. A rich compilation of novel electroactive materials toward urea electrosynthesis, together with critical thinking tips behind their synthesis and application, is presented in this section.

Recently, research in the field of urea electrosynthesis has been especially productive in the synthesis and engineering of new electrocatalysts for this specific reaction. Since the electroactive material must be able to simultaneously activate two species, namely, CO_2_ and NO_3_^−^, a double rational design is inherent for the development of new electrocatalysts. Zhou et al. have summarized the most important strategies to achieve the urea electrosynthesis: enhancing the activity of reaction sites, the synergistic effect and the atomically sizing have been pointed to be the most prominent [21]. In this text, primary emphasis is placed on tandem electrocatalysis, intended as two catalytic frameworks that cooperate to achieve the desired reaction [75, 76, 77]. Within this concept, DAC constitutes an excellent strategy for enabling tandem electrocatalysis, as two spatially proximate metal atoms can jointly act as the main catalytic center and deliver dual functionality. Nevertheless, DAC represents only one of several viable approaches to realizing tandem electrocatalysis and here the role of heterointerfacing, alloying and doping will also be treated, taking selected examples from the most recent literature. The UY rate and selectivity (or FE) of each material will be reported. A more extended list of the reviewed electrocatalysts is reported in Table 3.

**TABLE 3:** Comparison of different electrocatalysts performance recently used for the urea electrosynthesis with information about the cell, electrolyte, membrane, urea detection methods, and measured stability of the electrocatalyst.

Material	Cell	Electrolyte	Membrane	Detection methods	**UY, mmol h** ^**−1**^ **g**_**cat**_^**−1**^	Urea FE%	Potential, V vs. RHE	Measured stability, h	Reference
**Heterointerfaces**									
Cu‐In_2_O_3_/C	H‐cell	CO_2_ sat 0.1 M KNO_3_	Nafion 117	DAMO‐TSC/urease	62.9	33.27	−0.5	30	[59]
FeP _0.9_ S_2.9‐x_/Ag_2_S	H‐cell	CO_2_ sat 0.5 M KHCO_3_ + 75 mM KNO_3_	Nafion 117	Urease/HPLC	19.3	15.4	−0.7	50	[45]
P‐Cu/Fe_2_O_3_	H‐cell	CO_2_ sat 0.1 M KNO_3_	N/A	Urease/H‐NMR/HPLC	62.74	73.81	N/A	15	[58]
CuCH/Fe_2_O_3_−CFs	H‐cell	CO_2_ sat 0.1 M KHCO_3_ + 0.05 M KNO_3_	N/A	Urease/H‐NMR	29.8	78.7	−0.2	60	[78]
Cu/Cu_2_O	H‐cell	CO_2_ sat 0.5 KHCO_3_ + 0.05 KNO_3_	Nafion 117	DAMO‐TSC/H‐NMR	10.5	42.3	−0.3	10	[40]
Pd_2_Au_1_/RuO_2_	Flow cell	CO_2_ sat 0.2 M KHCO_3_ + 0.05 M KNO_3_	AEM	H‐NMR	73.5	75.6	−0.5	50	[79]
NiFe‐DAC‐PANI‐PIM	H‐cell	CO_2_ sat 0.5 M KHCO_3_ + 0.02 M KNO_3_	Nafion 117	DAMO‐TSC	27.8	75.3	−0.6	N/A	[80]
**Alloys**									
Sn_2_Cu	Flow cell	CO_2_ sat 0.5 M KHCO_3_ + 0.4 M KNO_3_	AEM	DAMO‐TSC	72.6	41.3	−0.52	60	[30]
CuZn‐40% PTFE	H‐cell	CO_2_ sat 0.1 M KHCO_3_ + 0.05 M KNO_3_	Nafion 117	DAMO‐TSC	3.6	49	−0.5	10	[62]
CuCo@N‐MHCS	H‐cell	CO_2_ sat 0.1 M KHCO_3_ + 0.05 M KNO_3_	N/A	DAMO‐TSC/urease	21.8	32.8	−0.55	10	[81]
PdCuCoZn MEA	H‐cell	CO_2_ sat 0.5 M KHCO_3_ + 0.02 M KNO_3_	Nafion 117	DAMO‐TSC	30.6	70.2	−0.6	20	[24]
PdAuCuIrCo HEA	H‐cell	CO_2_ sat 0.02 M KNO_3_	N/A	DAMO‐TSC/H‐NMR	52.4	22.57	−0.9	6	[54]
α‐CuZn	Flow cell	CO_2_ sat 0.2 M KHCO_3_ + 0.02 M KNO_3_	N/A	DAMO‐TSC/H‐NMR	60.0	28.7	−0.52	12	[34]
B‐PdIn IMene	H‐cell	CO_2_ sat 0.1 M KNO_3_		DAMO‐TSC	15.7	24.65	−0.5	12	[25]
**Doped materials**									
A‐In@Box	H‐cell	CO_2_ sat 0.1 M KHCO_3_ + 0.05 M KNO_3_	N/A	M‐DAMO‐TSC/urease	38.5	51.43	−0.45	50	[27]
W/CuO	Flow cell	CO_2_ sat 0.1 M KHCO_3_ + 0.1 M KNO_3_	Nafion 211	Urease	56.4	61.14	−0.7	100	[43]
Zn‐Fe_2_O_3_/Ov	H‐cell	CO_2_ sat 0.2 M KHCO_3_ + 0.1 M KNO_3_	Nafion 117	Urease/H‐NMR	124.5	62.4	−0.7	50	[67]
Bi‐In_2_O_3_	H‐cell	CO_2_ sat 0.1 M KNO_3_	Nafion 117	Urease/H‐NMR	39.6	80.2	−0.4	120	[82]
Ni@CeO_2_	H‐cell	CO_2_ sat 0.1 M KNO_3_	Nafion 117	Damo‐TSC/urease/H‐NMR	36.2	70.1	−0.5	8	[41]
Cu‐In_2_O_3_	Flow cell	CO_2_ sat 0.1 M KNO_3_	N/A	Urease/H‐NMR/DAMO‐TSC	83.2	47.3	−1.4	10	[83]
**DACs**									
CuIn_1.07_‐COF	H‐cell	CO_2_ sat 0.1 M KHCO_3_ + 0.05 M KNO_3_	Nafion 117	DAMO‐TSC/urease/H‐NMR	48.7	54.7	−0.6	10	[84]
Ga&Y/CNP	H‐cell	CO_2_ sat 0.1 M KHCO_3_ + 0.05 M KNO_3_	Nafion 117	Urease	41.9	22.1	−1.4	21	[33]
CuSn/CS‐1	H‐cell	CO_2_ sat 0.1 M KNO_3_	Nafion 117	Urease	55.8	79.27	−0.7	40	[69]
Ru_1_–Bi_1_/MnO_2_	Flow cell	CO_2_ sat 0.1 M KHCO_3_ + 0.1 M KNO_3_	AEM	Urease	101.5	74.6	−0.7	100	[53]
NiMo‐DASC	H‐cell	CO_2_ sat 0.1 KHCO_3_ + 0.05 KNO_3_	Nafion	DAMO‐TSC	11.3	31.8	pulsed −0.5/−0.7	20	[85]
PCOF‐34‐Fe	Flow cell	CO_2_ + 0.1 M KHCO_3_ + 0.1 M KNO_3_	Nafion 117	DAMO‐TSC/urease/H‐NMR	136.6	51.3	−0.6	20	[23]
CuPPc‐4.0	H‐cell	CO_2_ sat 0.9 M KHCO_3_ + 0.1 M KNO_3_	Nafion 117	Urease	460	26.1	−1.3	3.7	[46]
Cu_2_B/C	H‐cell	CO_2_ sat 0.1 M KHCO_3_ + 0.1 M KNO_3_	Nafion 211	DAMO‐TSC	22.9	64.9	−0.4	200	[86]
Cu–In DAC	Flow cell	CO_2_ sat 0.1 M KHCO_3_ + 0.01 KNO_3_	PEM	DAMO‐TSC	19.8	63	−0.37	20	[87]
Ni‐PDA@g‐C_3_N_4_	H‐cell	CO_2_ sat 0.1 M KNO_3_ + 0.1 M K_2_SO_4_	Nafion	DAMO‐TSC/HPLC/urease	19.8	18.03	−1.3	5	[88]
In_1_‐MoB_2_	Flow cell	CO_2_ sat 0.1 M KHCO_3_ + 0.1 M KNO_3_	Nafion 117	Urease	56.3	73.5	−0.6	10	[89]
**Others**									
Cu‐MOF‐CQD	Flow cell	CO_2_ sat 0.1 M KHCO_3_ + 0.04 M KNO_3_	AEM	DAMO‐TSC	4.3	18.5	−0.5	5	[26]
C‐Cu‐A_4_	H‐cell	CO_2_ sat 0.9 M KHCO_3_ + 0.1 M KNO_3_	Nafion 117	Urease	890	45.4	−1.2	2.5	[29]
ZnAl‐LDH	H‐cell	CO_2_ sat 0.1 M KHCO_3_ + 0.01 KNO_3_	Nafion 211	Urease	1.35	4.37	−0.7	5.5	[90]
CNT‐O	Flow cell	CO_2_ sat 0.1 M KHCO_3_ + 1000 ppm KNO_3_	Nafion 211	DAMO‐TSC	1.7	10	−0.6	28	[68]
Fe acs	H‐cell	CO_2_ sat 0.1 M KHCO_3_ + 0.25 M KNO_3_	Nafion 117	Urease	37.2	39.8	−0.84	58	[91]
Cu_3_Mo_2_O_9_	Flow cell	CO_2_ sat 0.1 M KNO_3_	Nafion 117	Urease	177	40	−0.8	24	[50]
CuAC–CuSA@NM	H‐cell	CO_2_ sat 0.1 M KHCO_3_ + 0.05 M KNO_3_	N/A	Urease	42.4	N/A	−1.3	11.1	[92]
BiVO_4_@MIL‐5	H‐cell	CO_2_ sat 0.1 KNO_3_	N/A	DAMO‐TSC	47.7	23.5	−0.9	10	[39]
CoCu NS	H‐cell	CO_2_ sat 0.1 M KHCO_3_ + 0.05M KNO_3_	Nafion 117	Urease	24.5	46.8	−0.7	10	[38]
Cu_5_‐PPF	H‐cell	CO_2_ sat 8 mM KNO_3_ + 0.2 M K_2_SO_4_	Nafion 117	DAMO‐TSC/HPLC	1.8	61.6	pulsed −0.5/−0.7	11	[47]
Cu‐MoS_2_/MoO_2_	Flow cell	CO_2_ sat 0.1 M KNO_3_	AEM	Urease	117.5	28.8	−0.6	68	[93]

Specifically, we have reported the UY rate, also simply named yield, with the value and units as they are in the referenced paper. Instead, the units in Table 3 have been unified for better and straightforward comparison. Anyway, Equation (4) reports the conversion factor between the two most used units.

(4) $$UY\;\left(\mathrm{\mu g}\;h^{-1}\;{\mathrm{mg}}_{\mathrm{cat}}^{-1}\right)=60.06\cdot \;UY\;(\mathrm{mmol}\;h^{-1}g_{\mathrm{cat}}^{-1})$$

### 4.1 Heterointerfacing

The creation of heterointerfaces has been addressed as a powerful strategy to boost electrocatalytic reactions through different effects. Li et al.*.* have summarized these effects in i) the generation of the space charge region; ii) the built‐in electric field effect; iii) the synergistic effect; iv) lattice strain effects and v) geometric effects [94]. Specifically, for the CO_2_RR, but also extendable for other reactions, Li et al. have also considered the spillover effect [95]. In the context of urea electrosynthesis, interfacing two different components can be an effective methodology to activate the CO_2_RR on one of the two materials and the NO_3_RR on the other, while the C—N coupling steps necessary for urea production can happen at the interface. Synergist effects, spillover effect, and geometric effects are highlighted as the most prominent in play. Indeed, the interface itself can be a more efficient place for the C—N coupling step than the single materials (synergy). Also, one surface of the two can be more effective for the C—N coupling step in the way that certain intermediates are spilled there from the other material (spillover). Ultimately, the geometry or shape of the materials is vital to maximize and optimize active sites exposure and the heterointerface area. Electronic effects have been also reported especially when charge redistribution between two metal atoms happens at the heterointerface and optimize certain intermediates adsorption. On the other hand, electric effects such as the built‐in electric field have not been explored as functional effects to improve urea production performance. Anyway, multiple examples are still present in the literature, especially concerning the other effects mentioned. Xu et al. have produced a Cu‐In_2_O_3_/C composite composed of rod‐shaped carbon matrix and irregular nanoparticles (NPs) of Cu and In_2_O_3_, synthesized by annealing a Cu–In MOF in N_2_/H_2_ atmosphere. The material has shown a UY and FE of 3779.26 μg h^−1^ mg_cat_^−1^and 33.27% at −0.5 V versus RHE, respectively. Mechanistic studies attributed the activity of the material to an optimal *d*(Cu)–*p*(O)–*p*(In) orbital interaction at the interface, where *NH_2_ formed on the Cu site and *CO formed on the In site can easily couple [59]. Liu et al. have prepared a 2D/0D FeP_0.9_S_2.9–*x*_/Ag_2_S heterostructure by in situ S‐extraction by AgNO_3_ from FeP_0.9_S_2.9_ nanosheets (NSs). This material has reached a UY of 1160.9 μg h^−1^ mg_cat_^−1^ and a FE of 15.4% at −0.7 V versus RHE, where FeP_0.9_S_2.9–*x*_ NSs serve as NO_3_^−^ activator while Ag_2_S promotes the CO_2_ to CO conversion [45]. Deng et al. have engineered Cu/Fe_2_O_3_ NPs by P‐doping, denoted as P‐Cu/Fe_2_O_3_, which has achieved a UY of 62.74 mmol h^−1^ g_cat_^−1^ and a FE of 73.81% at −0.68 V versus RHE. P‐doping was revealed to decrease energy barriers for *CO and *NO production on Cu and Fe_2_O_3_, respectively, but also to enhance *H coverage [58]. Song et al. have grown copper carbonate hydroxide and iron oxide on carbon fibers (CuCH/α‐Fe_2_O_3_‐CFs) by a hydrothermal method, which reached a UY and FE of 1794 μg h^−1^ mg_cat_^−1^ and 78.7%, respectively. α‐Fe_2_O_3_ promotes NO_3_^−^ to *NO conversion while CuCH serves as CO_2_ activator by asymmetric bridging‐adsorption of CO_2_ on CO_3_^2^‐Cu^2+^ dual site. Indeed, the carbonate interacts with the C atom in CO_2_ while copper interacts with the Oatom, creating an electron‐deficient C atom on CO_2_ which is easily attacked by *NO [78]. Wang et al. have synthesized oxygen‐vacancy‐rich Co_3_O_4_‐CuO nanowires on Cu foam (Co_3_O_4_‐CuO NWs/CF) by a hydrothermal method followed by annealing at 300°C. The material has shown a UY of 1.12 mg cm^−2^ h^−1^ and a FE of 35.89%, whereas control experiments on CuO/CF and Co_3_O_4_/CF have demonstrated the ability to better activate the NO_3_RR and the CO_2_RR, respectively [96]. Dai et al. have achieved urea electrosynthesis at the heterointerface of Cu/Cu_2_O microparticles, generated by in situ electrochemical reduction of commercial bulk Cu_2_O, which produced urea with a UY of 632.1 μg h^−1^ mg_cat_^−1^ and a FE of 42.3% at −0.3 V versus RHE. It was shown that the urea electrosynthesis performance is initially positively correlated with the interface content, but then saturates and decreases for higher Cu content than the optimal level. ATR‐FTIR and DFT calculations have established ET from Cu_2_O to Cu, which facilitates the formation and coupling of *CO and *NOH [40].

**FIGURE 7:**
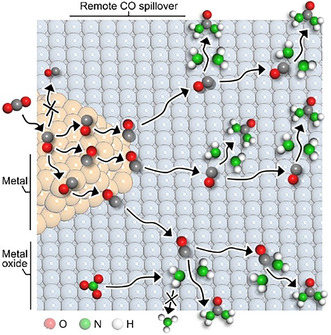
Schematic diagram of the design of remote CO* spillover improved tandem urea electrosynthesis on Pd_2_Au_1_/RuO_2_. This image was taken from [79] with permission, Copyright 2025, German Chemical Society (GDCh).

Li et al. have studied the activity of RuO_2_‐supported Pd_2_Au_1_ (Pd_2_Au_1_/RuO_2_), which has shown a UY of 73.5 mmol h^−1^ g_cat_^−1^ and a FE of 75.6%. The minimized work function difference between the two components promotes the *CO spillover from Pd_2_Au_1_ to RuO_2_, where it can be coupled with *NH_2_ (Figure 7) [79].

Also, a polymer heterointerface approach has been reported for urea electrosynthesis, in which the working principle is quite different compared to inorganic‐based materials. Indeed, Wang et al. have achieved high urea activity and selectivity using an ordered coating of bilayer polymers consisting of an upper hydrophobic polymer (PIM layer) to inhibit HER and favor CO_2_ diffusivity and a lower polymer layer (PANI layer) to increase local CO_2_ concentration. Here, PIM is a polymer of intrinsic microporosity with pore size distributions of 4–9 Å, while PANI stands for polyaniline. This engineered microenvironment coupled with a NiFe DAC has reached a maximum UY of 1671.6 µg h^−1^ mg_cat_^−1^ at −0.6 V versus RHE and a maximum FE of 75.3% at −0.5 V versus RHE in the NiFe DAC‐PANI‐PIM architecture (written from the lower to the upper layer), improving the performances compared to NiFe DAC, NiFe DAC‐PANI, and NiFe DAC‐PIM [80].

### 4.2 Alloying

Alloying can be an effective alternative approach to create two or more catalytic functionalities in the same material. Zhang et al. have pointed out i) composition regulation; ii) morphology control; iii) defect engineering; iv) surface engineering; and v) strain engineering as the most important aspects to tune the electrocatalytic activity of alloy‐based materials [97]. For urea electroproduction, it seems that the research is still looking for the mix of the right metallic elements and their compositional regulation to activate carbon dioxide and nitrate on two different metal sites while simultaneously boosting their coupling and disfavor single molecule reduction products. Other effects are advised to be studied in the future. Also, as an inherent peculiarity of alloys, their periodic crystal structure is here highlighted as another important aspect of their synthesis. Indeed, different phases could differently affect the active site local environment, which usually strongly affects its electrocatalytic performance. Moreover, HEAs are an emergent class of materials in electrocatalysis, which have been rarely reported for the electrosynthesis of urea but have great potential. Indeed, their electronic tunability for optimized intermediates adsorption and activation and the synergistic interaction of multiple metal sites make these class of materials attractive for the C—N coupling reactions [98]. Different alloy‐based materials have been reviewed here for urea electrosynthesis.

**FIGURE 8:**
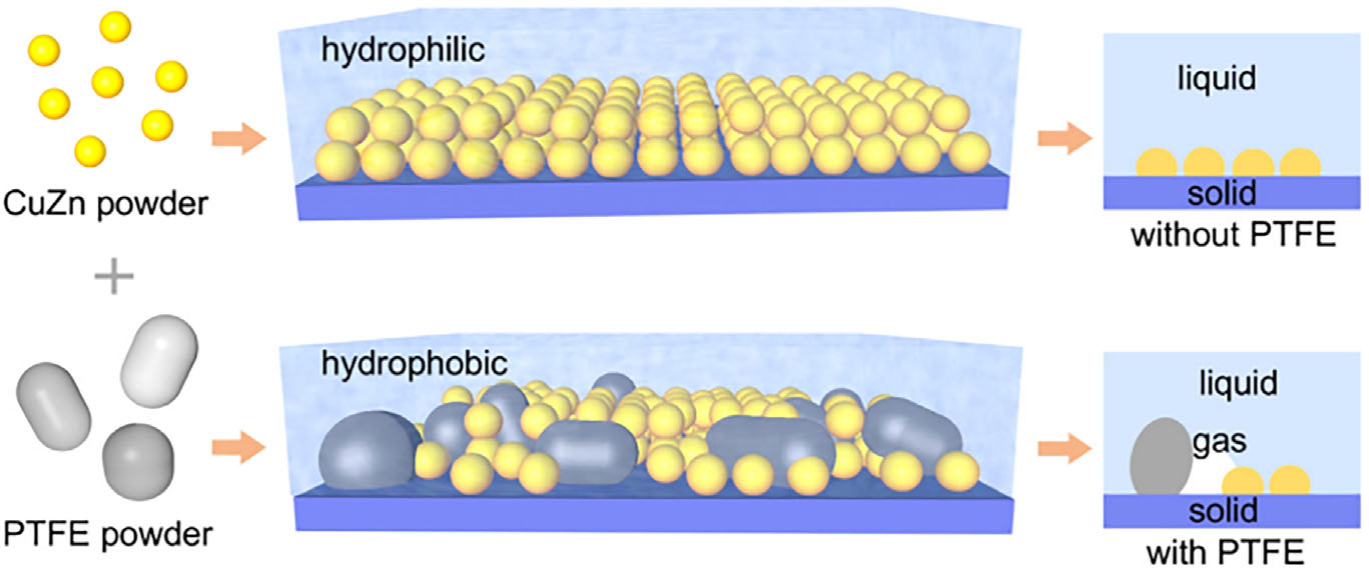
Schematic illustration of the fabrication route for various hydrophobic CuZn catalytic electrodes and their effect on the generation of a triple‐phase microenvironment. This image was taken from [62] with permission, Copyright 2026, Elsevier.

Feng et al. have engineered an atomic‐scale Mott–Schottky analogy in SnCu nanoalloy synthesized by in situ electrochemical reduction of Sn_*x*_‐CuO precursors. Among the various Sn:Cu ratios, Sn_2_Cu has shown the maximum UY and FE of 29.9 mmol h^−1^ g_cat_^−1^ and 46.7%, respectively, at −0.52 V versus RHE. Synchrotron radiation FTIR spectroscopy and DFT calculations have revealed electron‐enrichment of the Cu site, which optimizes adsorption energies for the *CO–*NHO coupling and suppresses HER [30]. Pei et al. have fabricated a hydrophobic triple‐phase catalytic interface by blending commercial CuZn alloy with PTFE particles. The 40% PTFE‐added CuZn alloy has reached a UY of 220 μg h^−1^ cm^−2^ and a FE of 49% at −0.5 V versus RHE compared to the 70 μg h^−1^ cm^−2^ and 8% of the PTFE‐unblended CuZn. The reason was attributed to the PTFE‐enhanced CO_2_ local concentration and NO_3_^−^ overreduction suppression (Figure 8) [62].

Yu et al. have designed and produced CuCo alloy NPs anchored to N‐doped microporous hollow carbon spheres (CuCo@N‐MHCS) to favor both selectivity and stability of pristine CuCo NPs. The prepared material has achieved a UY of 1312.3 μg h^−1^ mg_cat_^−1^ and a FE of 32.8% at −0.55 V versus RHE, whereas control catalysts, in situ FTIR, and DFT calculations have shown that Cu promotes *NH_2_ formation while Co produces *CO [81]. Zhou et al. have synthesized a quaternary PdCuCoZn medium‐entropy alloy metallene through the co‐reduction of metal acetylacetonate salts in the presence of W(CO)_6_ as a capping agent to afford the 2D structure. The medium‐entropy alloy achieved a maximum UY and FE of 1840 μg h^−1^ mg_cat_^−1^ and 70.2% at −0.6 V versus RHE. XAS and Bader charge analysis have revealed the electronegativity difference‐driven charge redistribution in the form Pd^δ−^ and Cu/Co/Zn^δ+,^ which ultimately favors *CO and *NH_2_ formation and stabilization, respectively [24]. Chen et al. have produced a HEA with a solid solution structure of formula PdAuCuIrCo, which has improved the C—N coupling (UY 52.43 mmol h^−1^ g^−1^ and FE 22.57% at −0.9 V versus RHE) compared to the MEI AuCuIrCo, which improved ammonia production. Theoretical calculations have shown a reduced *NO_2_ adsorption energy on the HEA and lower energy barriers for the C—N coupling steps [54].

Zhai et al. have investigated the different electrocatalytic activities of two brass phases, namely, α‐CuZn and β‐CuZn, supported on carbon nanotubes. α‐CuZn has been demonstrated to be the most urea active, with a UY of 60.0 mmol h^−1^ g^−1^ and a FE of 28.7% at −0.52 V versus RHE, attributed to a favored electronic structure (from COHP and PDOS analysis) and lowered energy barrier for the *CO_2_–*NO_2_ coupling [34]. Xu et al. have synthesized a B‐doped PdIn intermetallic bimetallene (B‐PdIn IMene) through a solvothermal method followed by postmodification with NaBH_4_ as B‐source. The B‐PdIn has displayed a UY of 943.61 μg h^−1^ mg_cat_^−1^ and a FE of 24.65% at −0.5 V versus RHE, outperforming the nondoped IMene [25].

### 4.3 Bulk and Surface Doping

Doping plays a crucial role in electrocatalysis by enabling precise tuning of the electronic structure of catalytic materials. By introducing heteroatoms or foreign elements into a host lattice, the local charge distribution and density of states can be significantly modified, leading to optimized adsorption energies of key reaction intermediates. This is particularly important for overcoming the limitations imposed by scaling relationships in conventional electrocatalysts. In the field of urea production, a commonly adopted strategy is to dope the surface of one material with another metal or nonmetal able to better activate the CO_2_RR or the NO_3_RR. Moreover, doping often induces lattice strain and defect formation, such as vacancies, which have been reported in this text as important atomic features that mediate in the urea electrosynthesis process. These structural distortions can also promote charge transfer and stabilize reaction intermediates. As a result, doped electrocatalysts typically exhibit improved activity, selectivity, and long‐term durability compared to their undoped counterparts [99, 100].

**FIGURE 9:**
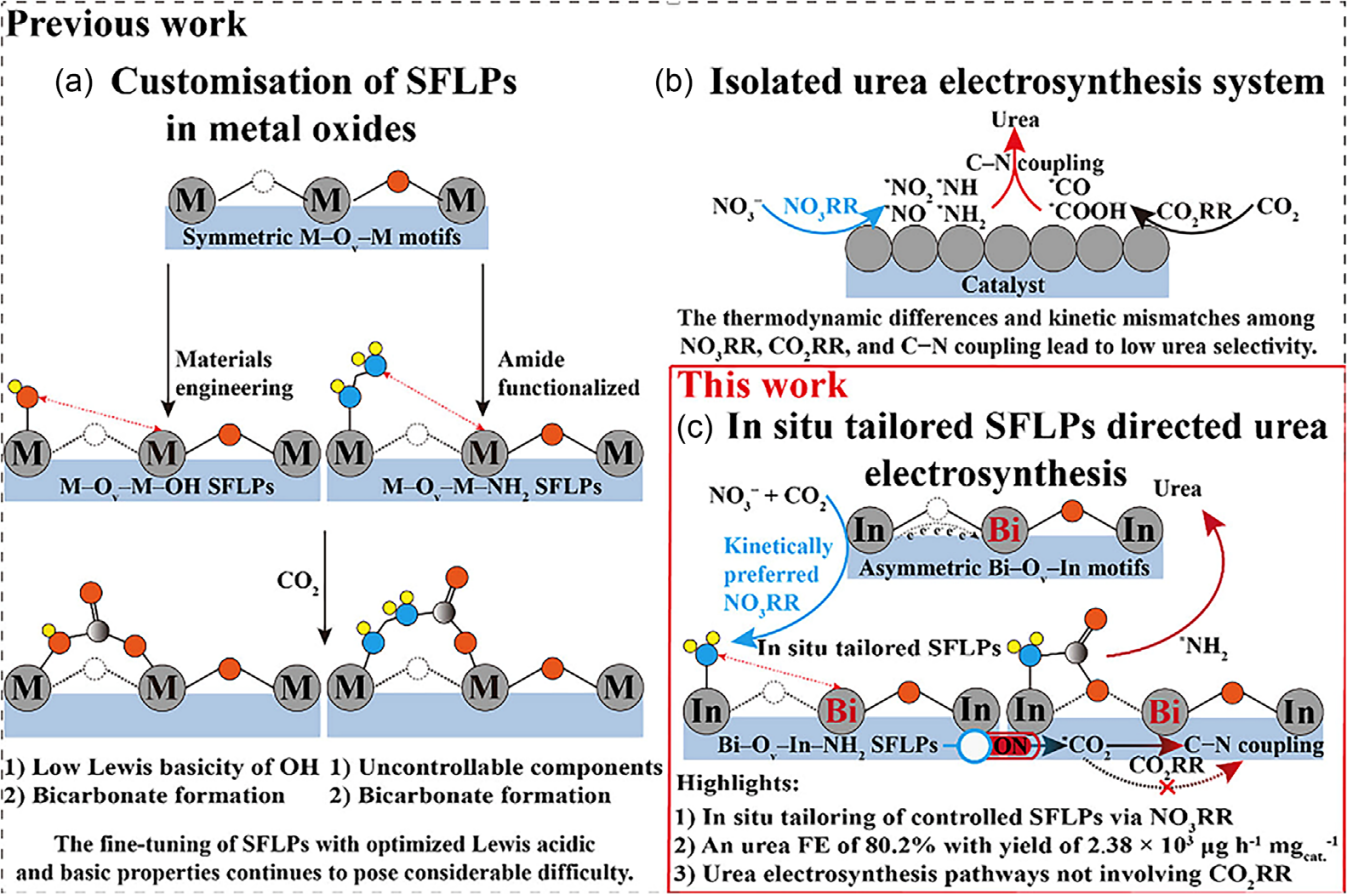
Rational design of Bi‐doped In_2_O_3_ which exploits SFLPs. Figure 9a depicts limitations at using a single‐metal oxide for SFLP‐driven intermediates coupling toward urea electrosynthesis while. Figure 9b points the inherent mismatch in term of therodynamics and kinetics for the CO_2_RR and NO_3_RR when using an isolted system. Figure 9c shows the reported improved methodology using a two‐metal oxide: In‐sites generates adsorbed *NH_2_ that is the holder of SFLP which can than trap CO_2_. On the other side, CO_2_ is effectively stabilized on Bi–O_v_–In motifs thanks to oxygen vacancies. This image was taken from [82] with permission, Copyright 2026, Wiley.

Here are some selected examples concerning the doping strategy for urea electrosynthesis. Liu et al. have developed amorphous In nanoparticles supported on boron oxides (A‐In@BO_*x*_) through a wet method followed by a freeze‐drying procedure. A‐In@BO_*x*_ has shown a UY of 2317.58 μg h^−1^ mg_cat_^−1^ and a FE of 51.43% at −0.45 V versus RHE and the activity was attributed to the undercoordination of In atoms, which hampers *NO_2_ generation (from ATR‐SEIRAS) while CO_2_ is calculated to be activated to *COOH [27]. Niu et al. have produced a W‐doped CuO nanowire arrays (W/CuO) which reached a UY of 56.46 mmol h^−1^ g_cat_^−1^ and a FE of 61.14% at −0.7 V versus RHE. DFT calculations have shown that W cooperates with Cu to enhance the C—N coupling between *CO_2_ and *NO_2_ but also helps to dissociate water into *H [43]. Song et al. have designed a Zn‐doped oxygen‐vacancy‐rich Fe_2_O_3_ catalyst (Zn‐Fe_2_O_3_/O_v_) with optimized adsorption energies of *CO, *OCNO, and *NOH containing asymmetric Zn–O_v_–Fe sites. Zn–Fe_2_O_3_/O_v_ has been produced by dipping the Fe_2_O_3_ into a Zn^2+^ solution (impregnation) followed by pyrolysis at 200°C in H_2_/Ar to create O_V_; the material has a UY of 7.48 mg h^−1^ mg_cat_^−1^ and a FE of 62.4% at −0.7 V versus RHE [67]. Li et al. have synthesized a Bi‐doped In_2_O_3_ with asymmetric oxygen vacancies by a solvothermal method followed by pyrolysis. The electrocatalyst has reached a UY of 2380 μg h^−1^ mg_cat_^−1^ and a FE of 80.2% at −0.4 V versus RHE. Its activity was attributed to the doping‐induced *NH_2_ extra‐stabilization on the In site in the In–O_v_–Bi moiety, which generated a surface frustrated Lewis pair (SFLP) able to directly couple with *CO_2_ (Figure 9) [82].

Liang et al. have prepared a single‐atom Ni anchored in the CeO_2_ lattice with oxygen vacancies (Ni@CeO_2–*x*_), which has delivered a UY of 2175.47 μg h^−1^ mg_cat_^−1^ and a FE of 70.1%. Mechanistic studies explained that O_v_ can mediate the high‐spin state Ni^2+^ to low‐spin state Ni^3+^ transition, which facilitates *NO‐to‐*NH_2_ conversion on the Ni^3+^ site while CO_2_ is activated to *CO on the Ce^3+^‐O site [41].

### 4.4 Dual‐Atom Catalysis

DACs are the “dual extension” of single‐atom catalysts which share many features and applications [101, 102]. Liu et al. have summarized the unique features of these catalysts, namely i) maximal atomic utilization, ii) highly dispersed active centers, iii) tunable electronic structure, iv) controlled coordination environment, and v) better catalytic performance compared to nondispersed active sites [102]. In 2025, DACs found the broadest application in the field of urea electrosynthesis. DACs have been defined by Roth‐Zawadzki et al. as “two metal atoms that are atomically close and serve as the main catalytically active site for the specific reaction” [103]. This is a particularly useful definition for the urea electrosynthesis, where two different active sites can promote the CO_2_RR and the NO_3_RR while maximizing atom efficiency (optimized site coordination and redox state) atom economy (low metal loading). Most importantly, the C—N coupling step can be sped up by the local proximity of two different active sites while their optimized compositional ratio can enrich the surface of CO_2_RR intermediates (sluggish kinetics) than NO_3_RR intermediates (faster kinetics). Recent reviews on the development of DACs for the CO_2_RR, the ammonia electrosynthesis, and the urea electrosynthesis have been published that could interest readers and highlight synthetic simplicity, atomic‐level structural control, and noticeable performances of this class of materials [103, 104, 105]. Here, we report some selected examples of engineered DACs for urea electrosynthesis, which mostly highlight the importance of the two‐atom choice as CO_2_ and NO_3_^−^ activators to the synergistic boost urea production and their characterization by XAS. Indeed, XANES and EXFAS are underscored as powerful tools to ultimately reveal redox state and coordination of atomically dispersed materials.

**FIGURE 10:**
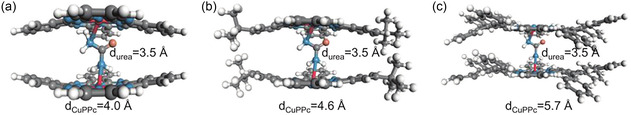
Schematic diagrams of matching interlayer diatomic Cu sites (*d*_CuPPc_) and molecular urea (*d*_urea_ = 3.5 Å), with atomic color scheme Cu (red), C (gray), N (cyan), O (orange), and H (white) for CuPPc with interlayer space of (a) 4.0 Å, (b) 4.6 Å, and (c) 5.7 Å, respectively. This image was taken from [46] with permission, Copyright 2025, German Chemical Society (GDCh).

Wang et al. have prepared a Cu‐In Covalent Organic Framework (CuIn‐COF) by post‐metalation of the imine‐linked COF with the relative metal salts. EXAFS analysis revealed the presence of Cu‐In (2.2 Å) Cu‐O (1.42 Å) and In‐N (1.62 Å). CuIn_1.07_‐COF has shown the highest ureal yield of 2924.4 μg h^−1^ mg_cat_^−1^ and FE of 54.7% at −0.6 V versus RHE. ATR‐SEIRAS and DFT‐based calculations have suggested *NH_2_ generation on the In site which then couples with *CO_2_ on the In site [84]. Chen et al. have designed a Ga and Y supported on N, P‐codoped carbon substrate (Ga/Y‐CNP) because of the CO_2_RR and NO_3_RR ability of the two metals, respectively. The material, synthesized by a two‐step procedure consisting of impregnation of carbonaceous precursors and pyrolysis, has an EXFAS‐derived metal coordination environment of the type (N)(O)Ga–Y(N)_4_. UY of 41.9 mmol h^−1^ g_cat_^−1^ and FE of 22.1% at −1.4 V versus RHE has been measured, while mechanistic studies have suggested the first C—N coupling between *CO on Ga site and *NH_2_OH on Y site [33]. Wu et al. have fabricated a Cu and Sn DAC anchored on CeO_x_ NPs supported on SiO_2_ (CuSn/CS‐1), which has reached a UY of 55.81 mmol h^−1^ g_cat_^−1^ and FE of 79.27% at −0.7 V versus RHE. XANES has revealed a mixed valence state of both elements, while STEM and EXFAS confirmed Cu and Sn atomic dispersion in the CeO_*x*_, without detection of Cu—Sn bonds. It was shown that Cu mediates CO_2_ to *CO conversion while Sn *NH_2_ production [69]. Xing et al. have produced Ru_1_Bi_1_/MnO_2_ composed of mutually isolated Ru and Bi atoms anchored on MnO_2_, prepared by a wet method. The material has achieved a UY of 101.5 mmol h^−1^ g_cat_^−1^ and a FE of 74.6% at −0.7 V versus RHE, whereas theoretical calculations have shown that the CO_2_ activation to *CO happens on Bi while NO_3_^−^ conversion on Ru [53]. Hu et al. have synthesized Ni–Mo pairs embedded in N‐doped graphitic carbon by pyrolysis of a mixture of Ni(NO_3_)_2_, (NH_4_)_6_Mo_7_O_24_, glucose, and melamine at 800°C and subsequent washing with HCl, in which STEM and EXAFS analysis revealed a Ni–Mo distance of 2.3 Å. The electrocatalyst has reached a UY 11.3 mmol h^−1^ g_cat_^−1^ and a FE of 31.8%, pulsing the potential at −0.5 V versus RHE for NO_3_RR and −0.7 V versus RHE to promote CO_2_RR, overperforming Ni‐ and Mo‐single‐atom electrocatalysts. Mechanistic studies have revealed Ni's capability to activate CO_2_ into *CO, while Mo enables NO_3_^−^ conversion into NH species [85]. Gong et al. have fabricated an entangled Fe porphyrin in a COF structure (PCOF‐34‐Fe) by direct metalation of the imine‐based entangled COF. In this way, the researchers have realized a nanoreactor made by twin Fe sites with optimized spacing of 8.8 Å where one site enables CO_2_RR and the other the NO_3_RR. Finally, the optimized distance facilitates the C—N coupling, and PCOF‐34‐Fe has reached a UY of 20 mmol h^−1^ g_cat_^−1^ and a FE of 90% at −0.5 V versus RHE [23]. This last example evidences the possibility of a DAC even with the same nature and valence state of the transition metal in both active sites (Fe^3+^N_4_). In line with this procedure, Zhang et al. have produced orderly layer‐stacking copper polyphthalocyanine (CuPPc) with optimized 4.0 Å interlayer spacings which have exhibited superior activity, with a UY of 460.0 mmol h^−1^ g_cat_^−1^ and a FE of 26.1% at −1.3 V versus RHE. Experimental and theoretical analyses have revealed that the 4.0 Å cavity spatially aligns intermediates to match the urea molecular dimension (3.5 Å), thus facilitating C—N bond formation (Figure 10) [46].

One example of DAC exploiting the same metal but in different valence states has been presented by Lin et al. They produced a Cu_2_Sb mix‐valence intermetallic compound (Mv‐IMC) on a carbon support by carbon impregnation of the metal precursors in a freeze‐drying step, followed by annealing in H_2_ atmosphere. X‐ray photoelectron spectroscopy (XPS) and XAS analyses have revealed electron donation from Cu to Sb, generating both Cu^+^ and Cu^2+^ states. The Cu_2_Sb Mv‐IMC electrocatalyst has achieved a UY of 22.9 mmol h^−1^ g_cat_^−1^ with a FE of 64.9% at −0.4 V versus RHE. In situ analyses and DFT calculation results have shown that Cu^+^−Cu^2+^ dual site can optimize the C—N coupling between *CO and *NO, avoiding their overreduction [86].

Conclusively, the diverse classes of electroactive materials design strategies discussed (heterointerfaces, alloys, doped systems, and DACs) highlight the wide range of available approaches to tailor catalytic performance at the atomic level. Each approach offers distinct advantages in modulating electronic structure, redox state of the active state, optimizing intermediate binding and enabling cooperative effects that are not accessible in simpler systems. Notably, these material design strategies represent some of the most effective and widely explored directions in the latest research here reviewed, reflecting the growing emphasis on precise control of active sites at the atomic level. Collectively, they provide a versatile and powerful toolbox for advancing urea production electrocatalysis toward higher efficiency, selectivity, and mechanistic control

## 5 Detection Methods

Accurate quantification of urea represents one of the most critical yet challenging aspects in the study of urea electrosynthesis. The typically low production rates and low concentration, coupled with the presence of interfering species, background contamination, and high concentration electrolyte matrix effect, make reliable detection far from trivial with many results presented in the literature that raise numerous doubts.

In their reviews, Kohlhaas et al. and Wojtas et al. have underscored the intrinsic troubles related to urea detection in electrochemical systems [14, 106]. First, low concentrations in the order of 1–10 ppm (where ppm means mg_urea_ L^−1^, or 16.7–167 μM) should be addressed with sensitive analytical methods, with a low limit of detection (LOD). Second, the electrolyte itself represents an issue since it is a highly saline solution, which could damage analytical instrumentation or may induce significant matrix effects, compromising analytical reliability. Third, other byproducts of the urea electrosynthesis are normally present in the electrolyte, such as nitrites (NO_2_^−^), ammonia (NH_4_^+^), hydrazine (N_2_H_4_), or formate (HCOO^−^) which could directly interfere with the urea detection method. On the other hand, gaseous products such as H_2_ and CO do not usually interfere because of their volatility. The most commonly employed urea detection approaches are colorimetric methods based on UV–vis detectors operating in the visible region. However, these methods are prone to both qualitative and quantitative limitations arising from interfering byproducts, pH variations, storage conditions, and exposure to light. Owing to these drawbacks, alternative analytical techniques are preferable for reliable urea determination, including nuclear magnetic resonance (NMR) spectroscopy and high‐performance liquid chromatography (HPLC) coupled with spectrophotometric or mass spectrometric (MS) detectors. The most important interferents and suggested countermeasures are highlighted in Table 4. Also, Table 3 reports the detection methods applied in each reported study. As concerns the detection of liquid and gaseous byproducts, an extensive review is presented by Rizzo et al. in their supporting information [107].

**TABLE 4:** List of the most common interferents and countermeasures for the developed urea detection methods. This table was readapted from [106], Copyright 2025, Wiley and Sons Gmbh under CC BY 4.0 license (Creative Commons Attribution 4.0 International).

Method	Interferent and influence nature	Interference range (unit)	Suggested countermeasure
DAMO	Nitrite—complex influence	<5 ppm and >20 ppm	Modified DAMO with 3% or 5% accuracy up to 30 or 50 ppm NO_2_^‐^, respectively; solid‐phase extraction with selective ion‐exchange resin.
	Fe ^3+^—noticeable underestimation Cu^2+^, Zn^2+^, Ni^2+^, Ru^3+^—moderate underestimation	>20 ppm >20 ppm	Use a suitable chelating agent (e.g., EDTA); consider using a lower concentration of Fe^3+^
Urease	Formate—pronounced underestimation	>460 ppm	Solid‐phase extraction with selective ion‐exchange resin
	Ammonia— underestimation	>3.4 ppm	—
	Ethanol—underestimation	>460 ppm	—
	Nitrite—pronounced underestimation	>0.1 M	—
	Cu^2+^, Fe^3+^, Zn^2+^, Mn^2+^—underestimation Ni^2+^, Ru^3+^—overestimation	>20 ppm	Use a suitable chelating agent (e.g., EDTA)
^1^H‐NMR	Water—overlap	Electrolyte	Water suppressing methods; concentrating analyte; accumulation of urea; high‐frequency NMR
	Formate—underestimation	>460 ppm	Solid‐phase extraction with selective ion‐exchange resin
^13^C‐NMR	HCO_3_^−^/CO_3_^2−^	Electrolyte	Control experiments
(UHP)LC‐(HR)MS	Inorganic salts—column clogging	—	Desalting via ion‐exchange resin; meticulous flushing of the column
	Solid particles—column/tubes clogging	—	Ultracentrifugation and/or filtration

### 5.1 Colorimetric Methods

Two colorimetric methods for the detection of urea are commonly used, namely, the DAMO‐TSC method and the urease method. The DAMO–TSC method relies on the reaction of urea with diacetyl‐monoxime (DAMO), after which a colored compound is generated. Its absorbance is correlated with urea concentration by a proper calibration curve. In the DAMO–TSC method, a given amount of the electrolyte is mixed with an acidic (H_2_SO_4_ + H_3_PO_4_) FeCl_3_ solution, DAMO, and thiosemicarbazide (TSC). Iron catalyzes the chromogenic reaction, while TSC is a color stabilizer [106]. The solution is then heated in boiling water for 15–20 min, cooled to room temperature and the absorbance is measured at 525 nm. It is widely reported that the DAMO–TSC method is susceptible to the effect of the heating time and light [108]. Mostly, the interference of nitrites can generate both false positives and false negatives [107]: this is the critical issue of this method since nitrites are often produced in parallel to urea. In 2023, Chen et al. proved a modified DAMO–TSC method (M‐DAMO–TSC) where nitrites are previously eliminated from the reaction mixture through their reaction with sulfamic acid, which reduces nitrites into gaseous dinitrogen [109]. Recently, Dai et al. have corrected the Ru^3+^, Pd^2+^, and Ag^+^ interference in the DAMO‐TSC method by adding Zn powder that reduces the noble metals, whereas Zn^2+^ has no effect [108]. The urease method exploits the reaction of the enzyme urease with urea at 37°C for about 40 min [106]. From this reaction, CO_2_ and ammonia are produced. Ammonia can be measured by the typical indophenol‐blue method [107, 110, 111] but also by ion chromatography [112]. Since ammonia is a byproduct of urea electrosynthesis, the ammonia content must also be measured before the urea analysis. Then, the urea‐derived ammonia equivalents can be obtained by subtraction and the urea content retrieved considering the 1 urea:2 ammonia stoichiometry of the urease reaction. The intrinsic criticalities of the urease method are the intrinsic use of an enzyme, whose activity is highly sensitive to temperature, pH, and salinity [70, 106]. Conclusively, the DAMO–TSC method and the urease method are often used because of their simplicity and cheapness, but are often poorly reproducible because of various sample preparation and treatments, which could differ from lab to lab, and of contaminations, which could be urea electrosynthesis by‐products or even metal cations leached from an unstable electrocatalyst.

### 5.2 NMR

**FIGURE 11:**
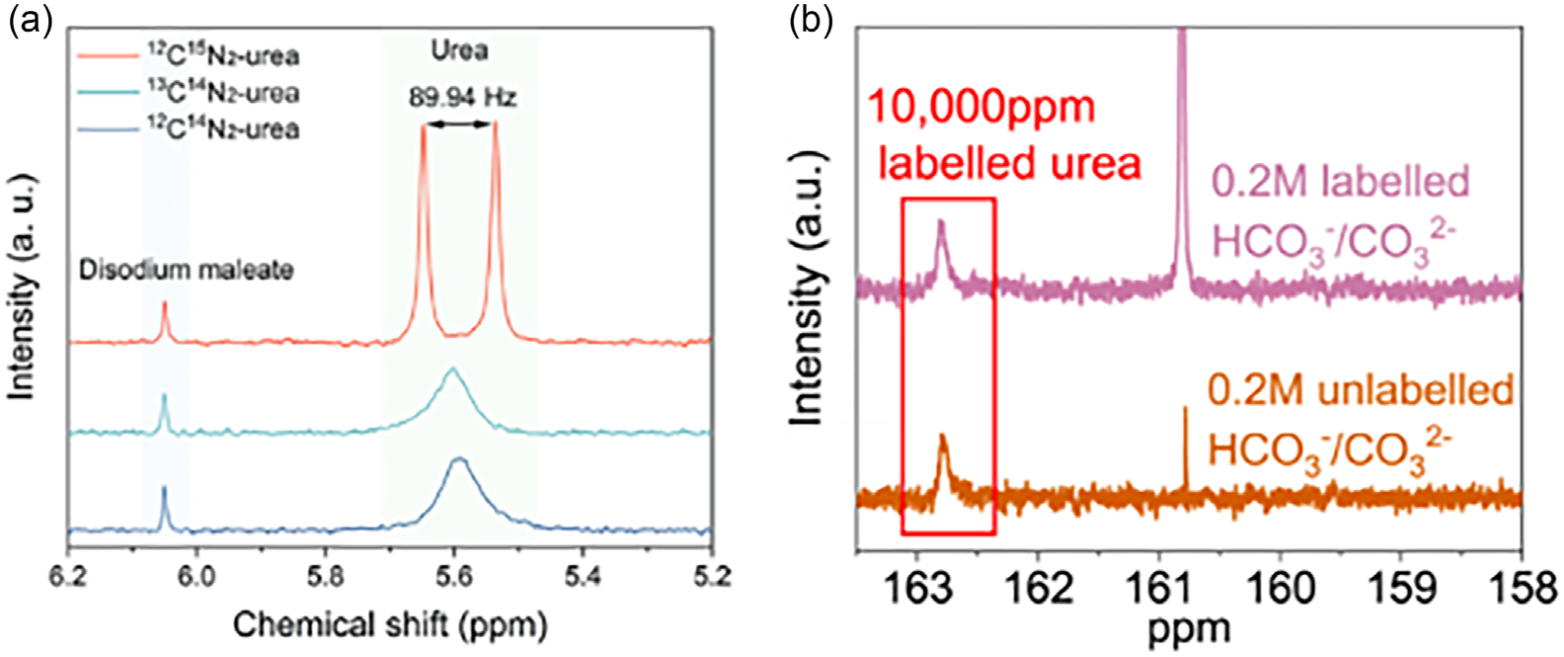
(a) 1H‐NMR spectra of ^12^C^15^N_2_‐urea (red), ^13^C^14^N_2_‐urea (turquoise), and ^12^C^14^N_2_‐urea (blue). (b) ^13^C‐NMR spectra of unlabeled urea and ^13^C‐labeled (purple) or unlabeled (orange) HCO_3_/CO_3_^2‐^ electrolyte. Image (a) was taken from [112], Copyright 2025, Wiley and Sons Gmbh under CC BY 4.0 license (Creative Commons Attribution 4.0 International). Image (b) was taken from [116], Copyright 2023, Cell Press Journal under CC BY 4.0 license (Creative Commons Attribution 4.0 International).

NMR spectroscopy is applied for the identification and quantification of urea. Wojtas et al. have recently summarized the main NMR methods for urea detection in electrochemical systems [106]. Normally, ^1^H is exploited, but ^15^N can also be indirectly assessed because of its magnetic influence on ^1^H [112]. The first property of an NMR spectrometer is the magnetic field intensity *B*_0_. The signal‐to‐noise ratio of an NMR experiment is proportional to *B*_0_^*3/2*^. In the case of urea, a small concentration (1–10 ppm), the right practice is to use spectrometers equipped with magnets stronger than 14 T (corresponding to 600 MHz ^1^H Larmor frequency), which, today, are accessible to most users [7]. In a typical ^1^H‐NMR experiment to quantify urea, the electrolyte and the deuterated agent, usually deuterated dimethylsulfoxide (d_6_‐DMSO) or deuterated water (D_2_O), are mixed in the NMR tube in a precise proportion. The mixture is then analyzed at the NMR spectrometer with the water suppression option. Then, the area related to the urea proton signal, which appears around 5.6 ppm, is integrated and correlated to urea concentration using a calibration curve. The good practice of quantitative NMR prefers the use of an internal standard [112, 113]. Anyway, not all groups use an internal standard, only relying on absolute integrated areas. Other groups use maleic acid or DMSO as an internal standard, to which the urea protons integrated area is referred [114]. Also, the different number of protons between the internal standard and urea must be considered, according to Equation (5).

(5) $$\frac{A_{\mathrm{urea}}}{A_{\mathrm{IS}}}=\frac{C_{\mathrm{urea}}\cdot n_{\mathrm{urea}}}{C_{\mathrm{IS}}\cdot n_{\mathrm{IS}}}$$

where *A* is the area, *C* is the concentration, and *n* is the number of protons, while IS denotes the internal standard. Despite the internal standard signal number of protons being known, the number of protons in urea is usually set to 4, notwithstanding their exchangeable nature with water [115, 116], which could decrease the actual value, affecting the calibration curve. For more details about ^1^H‐NMR urea detection and sample preparation, we recommend the Supporting Information and related references of the Viewpoint by Ferri et al. [70]. Checking the presence of ^15^N and ^13^C is usually conducted for mechanistic studies but also for quantitative analysis. Indeed, ^15^NO_3_^−^ or ^13^CO_2_ can be added as N‐ and C‐feedstocks to check urea generation from nitrates and carbon dioxide, excluding any other pathway. The existence, and even the quantification, of ^15^N_2_‐urea is confirmed because the proton signal centered at 5.6 ppm is split into two lines with coupling constant J_15N−1H_ of about 90 Hz (Figure 11a) [112]. On the other hand, ^13^C does not interact magnetically with ^1^H because of two bonds distance. Consequently, as reported by Tiange and Voznyy, ^13^C‐NMR is required, and ^13^C‐urea shows a signal around 162–163 ppm, while ^13^C‐bicarbonate/carbonate appears around 160–161 ppm (Figure 11b). In this work, rigorous protocols for ^13^C‐NMR urea detection method are defined [117]. To conclude, NMR seems to be an essential tool for mechanistic studies and isotopic experiments but is far from being used as a routine technique for quantification, also considering the low sensitivity in the ppm concentrations and the sometimes difficulty to access a ≥600 MHz spectrometer.

### 5.3 High‐Performance Liquid Chromatography

The necessity to perform accurate urea detection is shifting the urea electrosynthesis community to integrate common colorimetric methods with HPLC, which is less user‐dependent and allows byproducts separation, also offering a very low limit of quantification. Ferri et al. have listed all the columns used in urea electrosynthesis studies, which are Luna 5 μm NH_2_, Shimadzu C18, Phenomenex NH_2_, Toso ODS80TM, Sequant ZIC‐HILIC, whereas the typical eluents are water/acetonitrile mixtures [70]. Urea detection after the HPLC column can be performed using a simple UV–vis detector by measuring the absorbance of the eluate in the range 190–210 nm [106]. However, urea only shows an edge in this spectral region rather than a peak [118], making its selective detection risky. Moreover, other species can absorb the UV light at these wavelengths, such as water, nitrates, and carbonates, which have a much higher concentration than urea itself [119]. Recently, Rizzo et al. have developed an HPLC/UV–vis method using a HILIC column eluted with a formic acid‐buffered water/acetonitrile solution and detection wavelength at 195 nm [107]. A fluorescence detector can also be implemented with HPLC, but urea needs to be derivatized to make it fluorescent. Indeed, in 2007, Clark et al. developed an HPLC/fluorescence method by derivatizing urea with xanthydrol, which reacts quickly with urea at room conditions in an acidic environment [120]. Despite the fluorescence detection has a benefit of an extremely high sensitivity to low concentrations [121], the absence of this instrumentation in most of the electrochemistry and electrocatalysis labs is probably the reason why this method is never applied. Finally, the use of a MS system has been explored by Yuan and Voznyy who have tracked the intensity of [2urea+H]^+^ (*m*/*z* = 121.1) in a total ion counting (TIC) chromatogram. The TIC intensity of this adduct has correlated with urea concentration better than that of [urea+H]^+^ [117]. Recently, Zhang et al. have proposed a method based on ultrahigh‐performance liquid chromatography with high resolution MS (UHPLC‐HRMS), that achieved a LOD down to 0.1 ppm [122]. At the end, the use of the HPLC to avoid false urea quantification seems to be the natural development at the crossing point between electrocatalysis and analytical chemistry, especially because of its ability to separate urea for the other components present in the electrolyte. However, the low UV absorption of urea limits accurate UV–vis detection. So, it is recommended to propose and optimize new methods based on fluorescence and MS detectors which, despite the initial cost investment, will be much more reliable than colorimetry.

Finally, the authors strongly believe that the “urea detection” issue currently represents a crucial bottleneck in urea electrosynthesis research. Addressing it requires a concerted and interdisciplinary effort, where electrochemists, materials scientists, and analytical chemists work in close synergy. Such collaboration is essential for the development of a robust “gold standard” method for urea quantification in electrochemical systems, able of accurately accounting for low concentrations, the presence of interfering species, and the complex effects of high‐concentration electrolytes. Only through this collective effort will it be possible to establish reliable benchmarks and accelerate progress in the field. Up to now, several measurements reported in the literature are not reliable and often the presence of interfering species, and the complex effects of high‐concentration electrolytes substantially increased the reported value of urea electrochemically produced.

## 6 Conclusions and Outlooks

In this review, the most essential information in the research activities carried out in the past 2 years (from 2024) in the field of the electrosynthesis of urea has been reported. As the field is substantially new, lots of room for the improvements and an increase in knowledge is expected.

• Attention has been devoted to the following aspects to which the authors recommend some possible new research lines and improvement strategies.

### 6.1 Reaction Mechanisms Investigation and Clarification

Despite the most fundamental pathways to achieve urea from NO_3_^−^ and CO_2_ have been envisaged, we believe that the development of in situ and operando techniques with higher spatial resolution, ideally at the level of the active sites, might be revolutionary to observe experimentally where each intermediate is formed. Indeed, this is normally assessed by adsorption energy calculations of a given intermediate on a specific active site or through free energy diagrams. Spatial and time resolution are probably the first principles today in the whole field of electrocatalysis, for a better understanding of reaction mechanisms, especially on complex surfaces. As a suggestion, spatial resolution on a complex surface composed of different active metals could be achieved by XAS or X‐ray photoelectron spectroscopy (XPS) which are able to probe spectroscopically the environment of a single metal by selecting its characteristic excitation. So, the analysis of XAS or XPS spectra collected in operando conditions could give insight into the intermediate's nature, coordination, and structural evolution of a specific metal or active site under investigation. On the other hand, time resolution is still challenging in electrocatalysis and limited to some seconds or minutes in common spectroscopic systems such as FTIR, Raman, XAS, or XPS. DEMS allows a very fast detection of volatile products because of the intrinsic speed of the MS detector, enabling in‐time detection. As reported by Timoshenko and Roldan Cuenya, a possibility to gather spatial and time resolution could be represented by quick X‐ray absorption fine structure (QXAFS), the “speeded” version of XAS, and other related pump‐probe techniques, which reach time‐resolution down to milliseconds. These methods rely on peculiar X‐ray scanning monochromators and very fast data acquisition systems but find few applications in heterogenous catalytic systems where macroscopic effects such as temperature, concentration, and pH gradients may influence the experiment [123]. Conclusively, it is strongly encouraged the scientific community to bring their operando techniques know‐how in the field of urea electrosynthesis which seems to be a perfect platform for the development of new operando experiments.

### 6.2 Electrolyte Effects

We have highlighted the role of the electrolyte as an important candidate to tune the selectivity and activity with the electrocatalyst. However, those studies that deal with some electrolyte effect always have at its center a new electrocatalytic material, usually of complex composition and morphology. We believe that electrolyte effects studies should also be performed initially on model catalyst systems, “simplest” as possible, to deepen the fundamental knowledge of this topic. Changing reactant concentrations, cation identity and using pH‐buffered electrolytes are the easiest‐to‐do but partially unexplored routes of urea electrosynthesis, which could give important insight into reaction mechanisms, kinetic parameters but also for potential industrial applications. On the other hand, the problem of selecting an “electrolyte benchmark” is relevant since urea performance comparison of very similar materials can be quite different when the electrolyte concentration is different. It is recommended to discuss among scientist and select an “electrolyte of compromise” with defined concentration of bicarbonates and nitrates. This route might help to compare different works coming from different scientists. As mentioned in the electrolyte section, this choice should first consider the different rate of CO_2_RR and NO_3_RR, the former slower than the latter, which generally requires a higher bicarbonate concentration to boost local CO_2_ concentration. Moreover, potassium cation could be taken as the reference cation because of the apparent boosted urea production performance.

### 6.3 Material Science to Synthesize Novel Electrocatalysts

As shown, the tandem electrocatalysis is necessary to better activate the CO_2_RR and the NO_3_RR, independently. However, we suggest the fabrication of electrocatalysts that also have an engineered spatial arrangement, at least in terms of distances between the active sites, which could favor the C—N coupling in a sort of nanoreactor. Nonetheless, right proportions between the active sites seem to be a relevant parameter since the different kinetics of the CO_2_RR and the NO_3_RR, which influence intermediate coverage, ultimately affect the C—N coupling rate. These two strategies could drive toward higher selective and active electrocatalysts. On the other hand, stability tests should be always performed under the relevant electrochemical conditions both in terms of urea electrosynthesis efficiency and material possible degradation. In the context of electrocatalyst benchmarking, common testing protocols should be used and pursued for different materials especially considering the electrocatalyst loading and work overpotentials. Surface area measurements by BET or ECSA should also be considered for correct comparisons. Also, stability criteria should be fixed in terms of current stability and performance stability. To this point, effects such as pH drift at the interface after prolonged operation may be determinant to alter product selectivity. However, these effects have not been addressed yet in the literature.

### 6.4 Common Analytical Methods for Quantifying Urea

Since colorimetric methods are still very popular, we strongly suggest the implementation of all the precautions possible, especially reporting in the supporting information of the paper the exact manipulation and treatment conditions of the sample. This seems to be the easiest way to reproduce results in other labs. Then, we hope for the development and implementation of HPLC methods coupled with fluorescence or MS detectors which are without doubt the safest in terms of accuracy, precision, and reproducibility compared to colorimetric methods. Here, developing and testing new derivatizing molecules which can easily and quickly react with urea and help and fasten the detection of urea is strongly encouraged. These species should be further accurately detected by fluorescence or MS detectors after a chromatographic separation from other interferents. Moreover, the highly concentrated electrolytes usually utilized in electrochemical cells still limit HPLC and MS instruments applicability. Consequently, many efforts should be devoted to the study of the protocol to limit possible salt‐induced damage to the analytical apparatus. For example, the use of a divert valve may be helpful. This valve allows the initial flow from the pump to be temporarily diverted, so that the water containing salts or other dissolved impurities does not enter the column. This prevents these unwanted components from affecting the separation or damaging the column material, ensuring that only the properly prepared sample reaches the stationary phase and not deposit in the MS ionization chamber. On the other side, NMR seems to be potentially useful only for isotopic experiments, but the necessity of high magnetic fields instruments primarily limits urea quantification in the ppm concentration range routinely.

Other important factors affecting the urea electrosynthesis performance have not been treated here but deserve future attention. The choice to conduct an urea electrosynthesis experiment in an H‐cell or in a flow cell is not trivial because cell geometry can influence both activity and selectivity, affecting mass transport phenomena and local electrolyte properties [70].

For new entry researchers, it is suggested to first achieve reproducible results in the H‐cell which is for sure the easiest apparatus to be built and operated. Moreover, it can be used for very different materials, making it suitable for benchmarking of newly synthetized materials. Then, more complex systems such as flow cells could be implemented. Flow cells tests pertain to the system compatibility in industrial‐like systems, where urea production performances depend not only by the electroactive material itself but of the global geometry which affects gas and mass diffusion in solution. Indeed, static mass transport conditions of the H‐cell may not be representative of real scaled‐up electrolyzers. However, it is easier to pursue the simple implementation of H‐cells for new prepared materials  because it is more affordable by most of the new electrocatalysis laboratory facing the new field of urea electrosynthesis.

The most common electrochemical methodology to perform the urea electrosynthesis is the chronoamperometry (constant potential) but industrial systems are usually designed to work under chronopotentiometry (constant current) since a constant current should ensure a constant yield rate of the desired product. However, constant current performance is practically never checked for new urea electrosynthesis active materials. Moreover, pulsed techniques seem to have potential in altering intermediate surface coverage and the structure of the electrochemical double layer, which has been shown in a few examples to improve urea electrosynthesis performance [47, 73, 85]. Also, coupling the cathodic urea electrosynthesis reaction with a proper anodic reaction is essential for possible future industrial applications. Indeed, normally the anodic compartment is charged with a nitrate and bicarbonate solution (without CO_2_ bubbling), where the kinetically limited oxygen evolution reaction (OER) happens on metallic counter electrodes. Some examples which exploit kinetically easier anodic reactions are already present in the literature, for example, the hydroxymethylfurfural oxidation reaction (HMFOR) [43], the ammonia oxidation reaction (AOR) [53], the methanol‐to‐formate oxidation reaction [79], but also the waste polyethylene terephthalate to glycol acid oxidation reaction [59]. Notably, the anodic reaction can generate another value‐added product, with significant economic advantages in the scale‐up systems. Anyway, the anodic reaction could produce some byproducts which, by membrane crossover, could affect the cathodic urea electrosynthesis reaction, both positively (e.g., refurnishing protons) or negatively (e.g., poisoning active sites). The study of new counter reactions rather than OER especially in flow cells industrial like systems is encouraged to be explored. On the other hand, the use of the same nitrate/bicarbonate electrolyte in both the cathodic and the anodic chambers of the H‐cell seems to be suitable as benchmark for the screening of new materials performances, so keeping OER as the counter reaction. Lastly, poor attention has been given to the membrane choice, which normally is a proton exchange membrane (PEM) among which Nafion 117 is the most widespread because of its ability to easily conduct protons, highly necessary at the cathode. Anyway, anion exchange membranes (AEM) are also reported, which choice is probably based on the necessity to facilitate NO_3_^−^ transport. We suggest future research to investigate possible membrane effects in the context of urea electrosynthesis, which are actually unknown [124, 125].

Conclusively, many fundamental questions remain open across the entire workflow, from the rational design and preparation of electrocatalytic materials to the accuracy, robustness, and standardization of urea quantification methods. This broad landscape of unresolved challenges not only highlights the complexity of the process but also underscores the enormous potential for scientific progress. As a result, the field offers wide room for meaningful contributions, making it an exceptionally fertile ground for active and impactful research in the coming years.

## Conflicts of Interest

The authors declare no conflicts of interest.

## Data Availability

The data that support the findings of this study are available from the corresponding author upon reasonable request.
